# QTL Mapping of Combining Ability and Heterosis of Agronomic Traits in Rice Backcross Recombinant Inbred Lines and Hybrid Crosses

**DOI:** 10.1371/journal.pone.0028463

**Published:** 2012-01-26

**Authors:** Zhen Qu, Lanzhi Li, Junyuan Luo, Peng Wang, Sibin Yu, Tongmin Mou, Xingfei Zheng, Zhongli Hu

**Affiliations:** 1 State Key Laboratory of Hybrid Rice, College of Life Science, Wuhan University, Wuhan, China; 2 Key Laboratory of Oil Crop Biology of the Ministry of Agriculture, Oil Crops Research Institute, Chinese Academy of Agricultural Sciences, Wuhan, China; 3 Hunan Provincial Key Laboratory for Biology and Control of Plant Disease and Insect Pests, College of Bio-Safety Science and Technology, Hunan Agricultural University, Changsha, China; 4 National Key Laboratory of Crop Genetic Improvement, Huazhong Agricultural University, Wuhan, China; Kyushu Institute of Technology, Japan

## Abstract

**Background:**

Combining ability effects are very effective genetic parameters in deciding the next phase of breeding programs. Although some breeding strategies on the basis of evaluating combining ability have been utilized extensively in hybrid breeding, little is known about the genetic basis of combining ability. Combining ability is a complex trait that is controlled by polygenes. With the advent and development of molecular markers, it is feasible to evaluate the genetic bases of combining ability and heterosis of elite rice hybrids through QTL analysis.

**Methodology/Principal Findings:**

In the present study, we first developed a QTL-mapping method for dissecting combining ability and heterosis of agronomic traits. With three testcross populations and a BCRIL population in rice, biometric and QTL analyses were conducted for ten agronomic traits. The significance of general combining ability and special combining ability for most of the traits indicated the importance of both additive and non-additive effects on expression levels. A large number of additive effect QTLs associated with performance *per se* of BCRIL and general combining ability, and dominant effect QTLs associated with special combining ability and heterosis were identified for the ten traits.

**Conclusions/Significance:**

The combining ability of agronomic traits could be analyzed by the QTL mapping method. The characteristics revealed by the QTLs for combining ability of agronomic traits were similar with those by multitudinous QTLs for agronomic traits with performance *per se* of BCRIL. Several QTLs (1–6 in this study) were identified for each trait for combining ability. It demonstrated that some of the QTLs were pleiotropic or linked tightly with each other. The identification of QTLs responsible for combining ability and heterosis in the present study provides valuable information for dissecting genetic basis of combining ability.

## Introduction

Since rice (*Oryza sativa* L.) is a staple food for more than half of the population worldwide, the ability to increase its yield potential would be a key factor in achieving the global rice requirement of 810 million tons in 2025 [1]. Please check to make sure this matches the first reference. Although there have been great achievements since the first intra-subspecific hybrid rice was developed in China in 1973 [2], the yield potential of hybrid rice has apparently reached a plateau due to the limited genetic diversity [3]. Several studies and breeding experiences have suggested that the identification of superior hybrids is important for the success of a hybrid breeding program [4]–[8]. However, field evaluation of all possible crosses among inbred lines requires a large number of crosses and extensive field tests, which are expensive and time-consuming. In practice only a small proportion of all possible experimental hybrids are evaluated in field trials. Therefore, efforts have been made to predict hybrid performance by using field data of related genotypes and molecular markers [8].

It is well known that certain inbred lines display hybrid vigour when crossed, in terms of heterosis. These vigorous lines are said to have favorable combining ability. Certain inbreds have the ability to combine well with testers, suggesting that these inbreds have good general combining ability (GCA). When an inbred combines well only in certain crosses, that means that it has good specific combining ability (SCA). The successful identification of superior hybrid combinations depends on the combining ability of the parents and the gene effects that are involved in the expression of quantitative and qualitative traits of economic importance [9]. General and specific combining ability effects are valuable genetic parameters in determining the next phase of breeding program.

Combining ability is conventionally estimated by diallel analysis. The NCII mating design (North Carolina mating design II) is the most powerful genetic design for analyses of combining ability and has been applied extensively to crop breeding programs [4]–[6], [9]–[10]. Shukla et al. [4] assessed the combining ability of 120 two-line crosses and their 34 parents including elite indica TGMS (Thermosensitive genic male sterile) lines. The study suggests tremendous prospects of combining improved japonica and tropical japonica germplasms having wide compatible genes with indica TGMS lines for exploitation of inter-subspecific heterosis. Joshi et al. [9] conducted diallel analysis in the F_1_ and F_2_ generations of hexaploid wheat. The study indicates that F_1_ hybrids showing high SCA and having parents with good GCA, into multiple crosses and/or bi-parental mating, or diallel selective mating could prove a worthwhile approach for further improvement of grain yield in bread wheat.

GCA and SCA of the maize grain yield interact strongly with environment [11]–[12]. These studies suggest that combining ability is a complex trait. With the advent and development of molecular markers, it is possible to dissect complex polygene systems into individual Mendelian factors. Many QTL analyses have been performed to tag the heterotic traits by using different types of molecular markers in several crops [8]–[9], [13]–[15].

With molecular marker (AFLP, RFLP, SSR etc.) plus the best linear unbiased prediction (BLUP), some scientists identified marker loci associated with quantitative trait loci for hybrid performance or specific combining ability (SCA) in maize and rice [4]–[8], [16]. These studies showed the high potential of joint analyses of hybrids and parental inbred lines for the prediction of performance of untested hybrids. In their studies, the combining ability of parents was measured by the molecular marker genotype of the parental lines. To date, no QTL mapping analysis of combining ability in rice has been reported. In the present study, we introduced a variant NCII mating design that produced three TC (testcross) populations by mating three photo-thermo-sensitive genie male sterile (PTGMS) lines with a BCRIL population. These four related populations were used to evaluate combining ability and heterosis of ten traits of agronomic importance through biometric and QTL analyses.

The objectives of the present study were to (i) assess the combining ability of TC hybrids and their parents and (ii) to detect and to evaluate the QTLs that control the combining ability and heterosis of these ten agronomic traits in the BCRIL and TC hybrids.

## Materials and Methods

### Plant materials

Two elite inbred lines, *Zhenshan97B* (as female parent) and *9311*, were crossed to produce F_1_ hybrids. The F_1_ hybrids were then backcrossed with *9311* (as female parent) to obtain BC_1_F_1_ plants. From the BC_1_F_1_ hybrids, 140 BC_1_F_8_ lines were developed through seven consecutive self-crossed generations. Based on a NCII mating design, three PTGMS lines (Hua893s, Hua888s, and Peiai64s) were selected as females and crossed with the BC_1_F_8_ and the two parental lines *Zhenshan97B* and *9311* to generate the hybrids. Among the three PTGMS lines, Hua893s and Hua888s were novel varieties. The selected line, Peiai64s, is the most popular PTGMS line in the current breeding system in China. Among the 140 BC_1_F_8_ lines, only 98 plant lines were successfully crossed with tester line Hua893s, Hua888s, and Peiai64s. Thus, three testcross (TC) populations were developed that comprised 98 Hua893s hybrids (Hua893s/BCRILs, TCP_1_), 98 Hua888s hybrids (Hua888s/BCRILs, TCP_2_) and 98 Peiai64s hybrids (Peiai64s/BCRILs, TCP_3_). The 98 BC_1_F_8_ lines were selfed to generate the BCRIL population.

### Phenotypic evaluation

The experiment was conducted in the summer of 2007 at the experimental field of Huazhong Agricultural University (Wuhan, China). All the 294 F_1_ TC lines, the 98 BC_1_F_9_ lines (corresponding to the parental lines of the TC hybrids) and the six hybrids derived from the crosses between the three PTGMS lines and the two parental lines (*Zhenshan97B* and *9311*) were planted in a randomized complete block design with three replications (plots). Each plot consisted of three rows, and each row had ten plants. Material sowing date was May 17. Twenty-five-day-old seedlings were transplanted to an experimental field in which the plants were spaced at a distance of 16.7 cm within each row and 26.7 cm between rows. The recommended agronomical practices for hybrid rice were applied in the experimental plots. The middle five plants in the central row of each plot were used for the data collection. The ten quantitative traits investigated were plant height (PH; in cm), heading date (HD; in days), tillers per plant (TP), panicle length (PL; in cm), full grains per plant (FGPP), seed setting rate (SS; as a percentage), grains per panicle (GPP), spikelets per panicle (SPP), grain density (GD), and grain yield per plant (YD; in g).

### Molecular markers and linkage maps

A linkage map of the BCRIL population was built by Wang *et al*. [17]. In their work 244 BC_1_F_8_ lines were used as the materials in the genotyping. This map comprised 122 polymorphic SSR markers and two InDel markers, and covered a total of 1349.3 cM of the 12 rice chromosomes with an average distance of 10.9 cM between adjacent markers.

### Data analysis and QTL mapping

#### Statistical analyses

Except for YD and HD, the means of the replications for each trait and for each population were used for the QTL and for other analyses. The YD was calculated from the mean yield of a sample of fifteen plants in three plots, and the HD was obtained from the first heading date of each line. Missing data for the investigated phenotypic values were compensated by using a mean value of observed data. Microsoft Excel 2007 was used for the series of statistical biometric analyses, including variance analysis of combining abilities, genetic parameters estimation of agronomic traits and the phenotypic correlation coefficients of traits between the BCRIL and TC populations, and among TC populations. The GCA variance effects of the parents and the SCA variance effects of the hybrids were estimated by the fixed model described by Mo [18].

#### QTL mapping

We assume that a quantitative trait is controlled by QTL Q, with two alleles (Q and q). This QTL located near by molecular marker M (with two alleles *M* and *m*) on the same chromosome. The recombination fraction between molecular marker M and quantitative trait locus Q is *r*. The genotypes of the two inbred lines (P_1_ and P_2_) and their F_1_ are *MMQQ*, *mmqq* and *MmQq*, respectively. The F_1_ produces four types of gametes *MQ*, *Mq*, *mQ* and *mq* with frequencies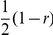, 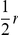, 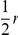 and 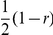. The F_1_ backcrossed to parental line P_2_ (*mmqq*) to produce BC_1_ hybrids. The BC_1_ hybrids self-crossed several generations to produce BCRIL (backcross recombinant inbred line) population. The genotype and genotype effect of molecular marker and QTL with two alleles at each locus in BCRIL population, TC population, Hmp data set, Sca data set and Gca data set were showed in Table 1.

**Table 1:** The genotype and genotype effect of marker and QTL for combining ability and heterosis with two alleles at each locus in BCRIL population.

	*MM*		*mm*	
Genotype in BCRIL population	*MMQQ*	*MMqq*	*mmQQ*	*mmqq*
Genotype effect in BCRIL population	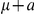	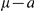	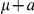	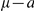
Genotype frequency in BCRIL population	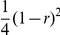	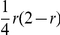	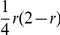	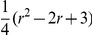

*MM* and *mm* denote the two genotype of molecular marker M; *QQ*, *Qq* and *qq* denote the three genotype of QTL; *r* represents the recombinant probability between molecular marker M and QTL; *μ*denote the overall mean value. *a* and *d* denote the additive effect and dominant effect, respectively; *q* and *p* denote the genotype frequency of QTL *QQ* and *qq* in tester, respectively (*p+q* = 1).

*When the genotype of QTL in tester is *QQ*,, and its genotype frequency is *q.*

# When the genotype of QTL in tester is *qq*,, and its genotype frequency is *p.*

§ When the genotype of QTL in tester is a mixture of *QQ* and *qq*, the genotype frequency of *QQ* and *qq* are *q* and *p*, respectively.

Using the additive-dominance model and in the absence of multiple alleles at the loci, the GCA and SCA effects derived from BCRIL and its related TC populations could be theoretically applied to the QTL analysis. The formulas for this theory were deduced as following (Table 1 and 2): (i) the GCA effect is equal to 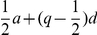 multiplied by a coefficient. The coefficient changes with the recombinant rate *r*. Where *a*, *d*, and *q* indicate the additive effect, dominant effect and the genotype frequency of the QTL *QQ* in tester, respectively. Only when the gene frequency of QTL *QQ* and *qq* in tester, *p* and *q*, are equal to 1/2, the effect of QTL detected by the Gca data set is additive; otherwise, it combines with both additive and dominant effects; (ii) the QTL effect in Sca data set is equal to *qd* or *pd* multiplied by a coefficient. The coefficient changes with the recombinant rate *r*. In other words, any QTL detected in the Sca data set shows only dominance. Obviously, except the dominant effect *d*, the efficiency of the QTL analysis of the Sca data set was also influenced by the gene frequency *q* or *p*. So the efficiency of the QTL analysis in SCA data set is clearly lower than that in the QTL mapping of Hmp data set (the effect of QTL detected in Hmp only influenced by the dominant effect *d*).

**Table 2:** The mean value of BCRIL population, TC populations, Hmp, Sca and Gca data sets with two alleles at each locus in BCRIL population.

Data set	Mean value
BCRIL population	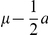
* TC population	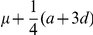
* Hmp data set	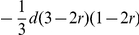
* Sca data set	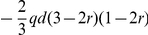
# TC population	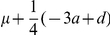
# Hmp data set	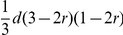
# Sca data set	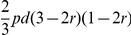
§TC population	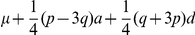
§Gca data set	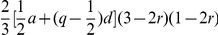

See footnotes of Table 1.

The above deduction could be easily extended to the case of multiple alleles at QTL loci in a NCII mating design when the base population is a BCRIL population (Table 3 and 4).

**Table 3:** The genotype and genotype effect of marker and QTL for combining ability and heterosis with multiple alleles at each locus in BCRIL population.

	*MM*		*mm*	
Genotype in BCRIL population	*MMQQ*	*MMqq*	*mmQQ*	*mmqq*
Genotype effect in BCRIL population	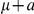	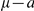	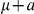	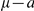
Genotype frequency in BCRIL population	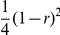	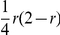	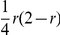	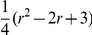

*MM* and *mm* denote the two different genotype of molecular marker M; *Q* and *q* denote two alleles of QTL in BCRIL population, Q_i_ (i = 1∼k) represents the multiple alleles of QTL in tester; *r* represents the recombinant value between molecular marker M and QTL; *μ*idenotes the overall mean value. *a*, *a*_1_ and *a*_k_ denote the additive effect of different allele; *g_i_* and *g_i_′* (1∼k) denote the genotypic value of the homozygote and heterozygote of QTL, respectively. 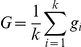, 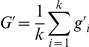.

*When the genotype of QTL is *Q*_1_ *Q*_1_ in tester.

# When the genotype of QTL is *Q*_k_ *Q*_k_ in tester.

**Table 4:** The mean value of BCRIL population, TC populations, Hmp, Sca and Gca data sets with multiple alleles at each locus in BCRIL population.

Data set	Mean value
BCRIL population	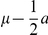
* TC population	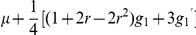
* Hmp data set	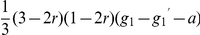
* Sca data set	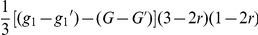
# TC population	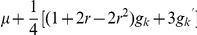
# Hmp data set	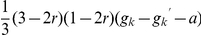
# Sca data set	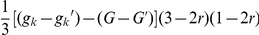
# Gca data set	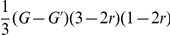

See footnotes of Table 3.

The QTL analysis was performed separately for the BCRIL and the seven independent data sets derived from these four related populations. The seven data sets were the midparental heterosis (Hmp) and SCA effects of three TC populations (including Hmp893s, Hmp888s, Hmp64s, Sca893s, Sca888s and Sca64s data sets) and the GCA effects of the BCRIL population (Gca-all data set). Hmp, SCA and GCA effects were estimated with the following equations: 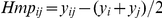; 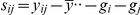; 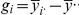. 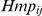 = midparental heterosis value of the TC hybrid between the parental lines BC_1_F_9_ line *i* and tester *j*; 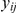 = phenotypic value of the TC hybrid between the parental lines BC_1_F_9_ line *i* and tester *j*; 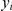 = the phenotypic value of BC_1_F_9_ line *i*; 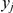 = the phenotypic value of tester *j*; 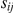 = SCA effect; 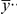 = overall mean; 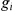 = GCA effects of BC_1_F_9_ line *i*; 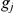 = GCA effect of tester *j* and 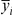 = mean performance of the three hybrids between BC_1_F_9_ line *i* and the three testers.

Analysis of the main-effect QTL (M-QTL) was conducted for each data set (including Gca, Hmp893s, Hmp888s, Hmp64s, Sca893s, Sca888s and Sca64s data sets) by composite-interval mapping using WinQTLcart 2.0 [19]. A LOD score of 3.0 was selected as the threshold for the presence of a main-effect QTL based on the total map distance and the average distance between markers. QTLs that were detected in different populations or for different traits were considered common if their estimated map position was within a distance of 20 cM [20], which is a common approach in comparative mapping [21]–[22].

The accuracy of the QTL analysis using the Gca data set was evaluated by a cross validation. Briefly, two of the three TC populations were selected to form the 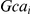 (*i* in {1..3}) data set for QTL mapping. This procedure was performed three times. All three pairwise combinations of the TC populations were subjected to QTL analysis. The results of the QTL analysis of these 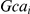 data sets are presented in the supplementary information files.

## Results and Discussion

### Performance of the populations

**Figure 1:**
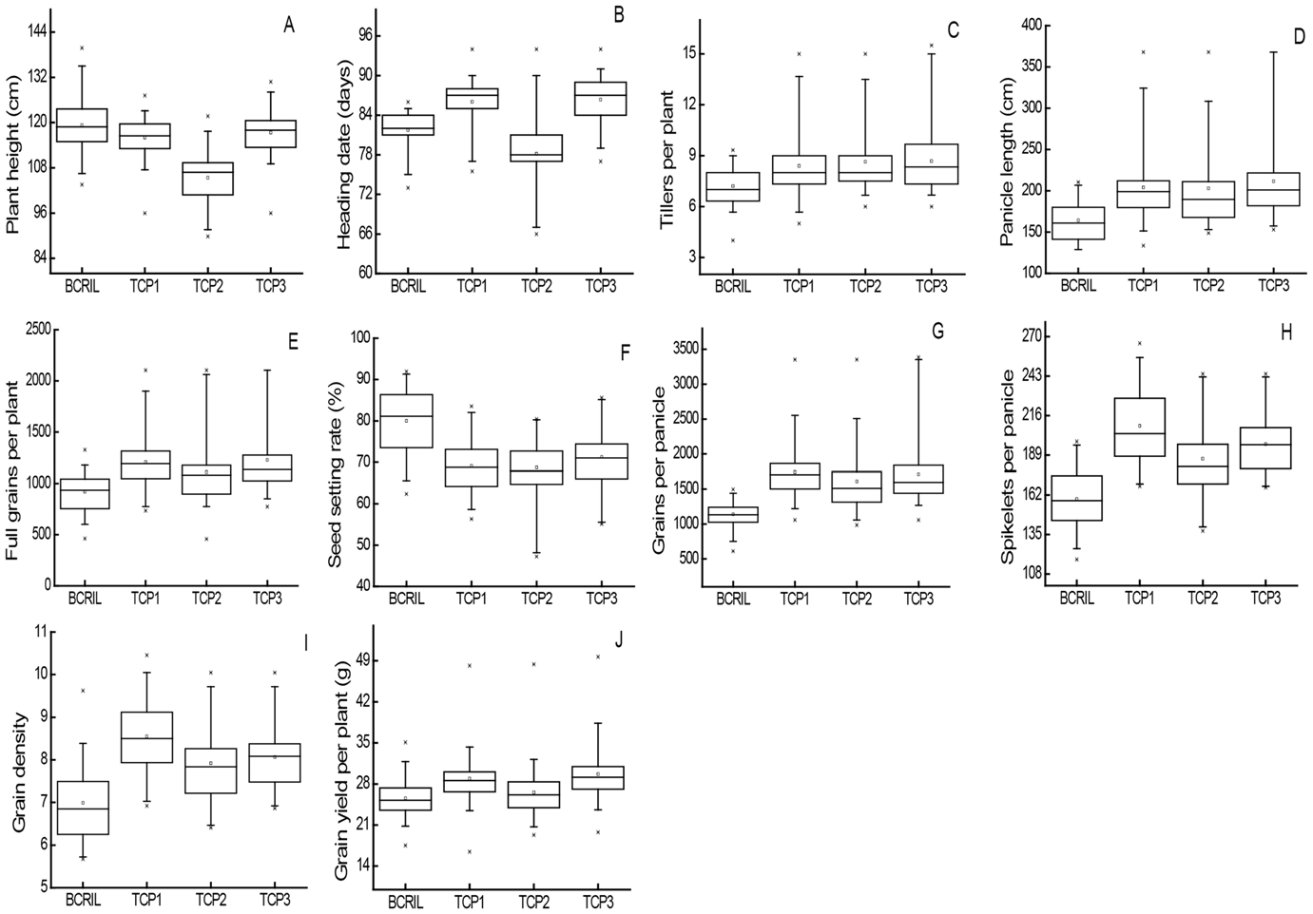
The means and ranges of ten quantitative traits measured in the BCRILs and their TC progenies.

The means and ranges of ten quantitative traits measured in the BCRILs and their TC progenies are shown in Fig. 1. The values of the ten traits varied widely in the BCRILs and their TC progenies and showed an approximately normal distribution.

The means of most of traits, except PH, HD and SS, in the TC populations were higher than the corresponding values in the BCRIL populations. The mean value of plant height and seed-setting rate of TC population were lower than those of the BCRIL population. In addition, the mean value of heading date of BCRIL population was lower than that of the Hua888sTC population, while higher than that of the other two TC populations. These results suggested that the three TC populations might possess a high level of special combining ability and heterosis.

### Relationships between trait values in the BCRIL and TC populations

Table 5 shows the correlation coefficients between the phenotypic values of the individual TC hybrids and the values of their paternal BCRILs, between the GCA effects and phenotypic values of the BCRILs, and between the SCA effects and Hmp in the TC hybrids for the ten investigated traits.

**Table 5:** Phenotypic correlation (*r*) coefficients for traits of agronomic importance between the mean trait values of BCRILs and TC hybrids.

	PH	HD	TP	PL	FGPP	SS	GPP	SPP	GD	YD
BCRIL and Gca	0.46**	0.56**	0.16	0.14	0.19	0.10	0.04	0.37*	0.25	0.01
BCRIL and TCP_1_	0.23	0.55**	0.16	0.03	0.10	0.10	−0.03	0.25	0.15	−0.06
BCRIL and TCP_2_	0.48**	0.38**	0.22	0.21	0.19	0.02	0.10	0.33*	0.22	−0.03
BCRIL and TCP_3_	0.36**	0.52**	0.06	0.12	0.20	0.11	0.02	0.31	0.29	0.10
TCP_1_ and HmpP_1_	0.77**	0.91**	0.96**	0.96**	0.94**	0.84**	0.98**	0.92**	0.88**	0.93**
TCP_1_ and ScaP_1_	0.35**	0.27*	0.44**	0.40*	0.26	0.54**	0.35*	0.67**	0.58**	0.74**
TCP_2_ and HmpP_2_	0.87**	0.97**	0.96**	0.96**	0.95**	0.89**	0.98**	0.92**	0.87**	0.93**
TCP_2_ and ScaP_2_	0.76**	0.84**	0.47**	0.48**	0.39*	0.69**	0.43**	0.70**	0.62**	0.75**
TCP_3_ and HmpP_3_	0.80**	0.92**	0.96**	0.97**	0.97**	0.88**	0.98**	0.86**	0.83**	0.94**
TCP_3_ and ScaP_3_	0.53**	0.30*	0.50**	0.58**	0.75**	0.70**	0.60**	0.86**	0.41*	0.80**
HmpP_1_ and ScaP_1_	0.55**	0.35**	0.43**	0.44**	0.31	0.47**	0.36*	0.70**	0.60**	0.70**
HmpP_2_ and ScaP_2_	0.70**	0.88**	0.43**	0.42**	0.38*	0.65**	0.39*	0.69**	0.59**	0.70**
HmpP_3_ and ScaP_3_	0.58**	0.37**	0.54**	0.57**	0.73**	0.62**	0.60**	0.76**	0.36*	0.73**

For a description of agronomic traits see materials and methods.

HmpP_1_, HmpP_2_ and HmpP_3_ idenote the Hmp values of TCP_1_, TCP_2_ and TCP_3_, respectively; ScaP_1_, ScaP_2_ and ScaP_3_ indicate as the Sca values of TCP_1_, TCP_2_ and TCP_3_, respectively.

*P< 0.05,

**P<0.01.

No significant correlation was detected between the means of the GCA effects and the phenotypic values of the BCRILs for most of the traits, except for PH, HD, and SPP (Table 5). Similar results were observed for the relationship between the phenotypic values of TC hybrids and their paternal BCRILs. However, the correlation coefficients among the phenotypic values of the BCRILs, the SCA effects and Hmp in the TC hybrids were significant for most of the evaluated traits.

### Variance analysis of combining ability

From Table 6, the mean squares due to GCA (BCRILs or PTGMS) and SCA (PTGMS×BCRIL) effects were found to be significant for most of the traits, with the exception of the GCA effect of TP (PTGMS) and PL (BCRILs and PTGMS) and the SCA effect of TP, PL, FGPP, and GPP, respectively. Thus, both kinds of gene effects were important in controlling the inheritance of most of the studied characteristics. In addition, the variance of the component estimates of GCA for all of the traits, except SS, was more than 59%. This suggests that genetic variation among crosses was predominantly additive, which differs from the results obtained in previous reports conducted in rice [4], [23]. It has been reported that the non-additive variance is important in respect of yield and its components [23]–[24]. The variance component estimates of SCA for SS, SPP and GD were more than 40% in the present study, which indicated that non-additive gene activity also plays an important role in the inheritance of these traits.

**Table 6:** Variance analysis of combining abilities and genetic parameters estimation of agronomic traits.

Trait	PTGMS	BCRIL	PTGMS×BCRIL	Error	V_gca_(%)	V_sca_(%)	V_gca_/V_sca_	V_A_	V_D_	*a.d.d*	*h* ^2^_N_(%)
PH	6196**	205**	39*	14	83.3	16.7	5.0	42.4	8.5	0.7	76.4
TP	6	11*	7	6	82.4	17.6	4.7	1.1	0.2	0.7	33.4
PL	1788	6013	4982	4680	74.7	25.3	2.9	296.0	100.5	0.8	15.1
FGPP	1304041**	327044*	175240	151954	83.4	16.6	5.0	38909.0	7762.2	0.6	40.0
SS	348**	157*	130*	53	47.4	52.6	0.9	23.0	25.6	1.5	34.7
GPP	1868366**	452416*	283583	234813	74.8	25.2	3.0	48356.2	16256.5	0.8	33.8
SPP	43402**	1940**	1177*	524	59.1	40.8	1.4	314.7	217.7	1.2	44.5
GD	33**	2*	2*	1	59.1	40.9	1.4	0.3	0.2	1.2	35.8

*P<0.05,

**P<0.01,

V_A_ (additive variance) and V_D_ (dominance variance) were always significant (*P*<0.05) for all traits. *a.d.d* and *h* ^2^_N_ indicate the average dominance degree and narrow-sense heritability, respectively.

For a description of agronomic traits see materials and methods.

The NCII analysis led to the estimates of V_A_ (additive variance) and V_D_ (dominance variance), which were always significant (*P*<0.05) for all of the traits (Table 6). The average dominance degree (*a.d.d.*) of several traits was less than 1, but this result was not obtained for SS, SPP and GD. This suggested an important contribution of over-dominance to the heterosis of these three traits.

The estimates of narrow sense heritability ranged from 15 to 76% in the studied characters (Table 6). The order of the narrow sense heritability of agronomic traits was PH>SPP>FGPP>GD>SS>GPP>TP>PL. Trait SPP, FGPP, GD, SS, GPP, and TP showed moderate heritability, which indicated that these characteristics could be improved by performing selections among the recombinants obtained from the segregating populations.

Marilia *et al*. [25] have suggested that a hybrid combination with a high performance *per se*, favorable SCA estimates, and at least one parent with a high GCA would tend to increase the concentration of favorable alleles, which is desirable for any hybrid breeding program. The TC hybrids assessed here possessed this combination of features. Table 7 shows the GCA effects of the different characteristics in the three PTGMS lines. Among the female parents, Peiai64s was the best general combiner for grain yield and a good combiner for most of the yield component characteristics. Hua888s was the poorest combiner for grain yield, and for most of the yield components. Hua893s was the best general combiner for GPP, SPP and GD. However, the parents that demonstrated the best performance *per se* were not the best general combiners (data not shown). This phenomenon may be due to the lack of a higher order additive interaction.

**Table 7:** Estimates of general combining ability effects for different characters in the three PTGMS lines.

PTGMS	PH	HD	TP	PL	FGPP	SS	GPP	SPP	GD	YD
H893s	3.1	2.5	−0.2	−2.1	25.8	−0.6	53.9	11.6	0.4	0.5
H888s	−7.5	−54	0.1	−3.2	−71.8	−1.0	−78.0	−10.9	−0.3	−1.8
Peiai64s	4.4	2.8	0.1	5.3	46.0	1.6	24.1	−0.8	−0.1	1.3

For a description of agronomic traits see materials and methods.

### QTL mapping for combining ability and heterosis

The genotype of molecular marker and QTL and their genotype frequency were deduced when the base population is BCRIL. Thus, it is feasible to conduct QTL mapping for combining ability and heterosis of the agronomic traits with this variant design. The genotype of molecular marker and QTL can also be deduced when the base population is RIL/DH (Table S1, Table S2, Table S3, and Table S4). This further confirmed that QTL mapping method could be successfully applied to combining ability and heterosis with different kinds of base population (RIL/DH/F_2_/BC/BCRIL) in NCII design.

Mo and Li [26] demonstrated that the homogeneity of gene frequencies between female and male parents under the augmented NCII design can be tested by a statistic method. It is well known that the gene frequency of RIL/DH/F_2_ population, *p* and *q*, equals to 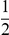. When the female parent is the RIL/DH/F_2_ population and the female and male parents are similar to each other in homogeneity of gene frequency, the gene frequency of male parent (test populations), *p* and *q*, must equal to 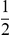. In the present study, the gene frequency of female parent BCRIL population does not equal to 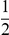 and the number of test populations was odd, so the homogeneity of gene frequency between female (BCRIL population) and male parents (test populations) were not tested.

Our analyses allowed the identification of several QTLs for each of the investigated traits. In total, 127 QTLs were identified for the ten traits evaluated in the eight data sets, and most of the individual QTLs explained more than 10% of the observed variation (Table S6 and Table S7). These results confirmed that combining ability and heterosis are polygenic phenomena. In the present study, we compared the QTLs that were detected in the BCRIL and Gca data sets and in the Sca and Hmp data sets in the TC population, respectively, to analyze the genetic basis of combining ability and heterosis.

### Main-effect QTL: *QTLs with an additive effect*

**Figure 2:**
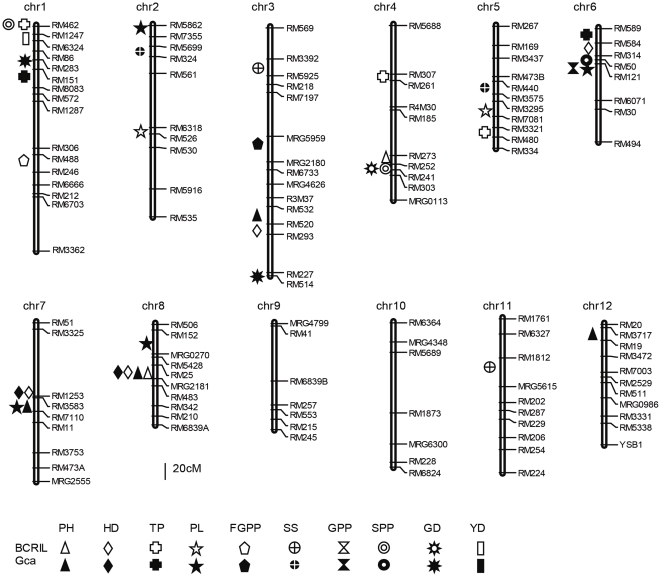
The QTLs detected in BCRIL and Gca data sets.

The QTLs detected in BCRIL and Gca data sets are shown in Table S6 and Fig. 2. Thirty-four main-effect QTLs that affected the ten traits in the two data sets were identified. Most of these QTLs could individually explain more than 10% of the variation.

PH: Five QTLs were detected. QTL *ph4* was identified in RM273-RM252 on chromosome 4 in the BCRIL data set. QTL *ph3*, *ph7* and *ph12* were detected in Gca data set. QTL *ph8* was found in both sets.

HD: Four QTLs were found. Two QTLs were detected only in BCRIL, and the other two (*hd7* and *hd8*) were found in both the BCRIL and the Gca data sets.

TP: Five QTLs were found in different intervals on chromosomes 1, 2, 4 or 6; each QTL was detected only in the BCRIL or GCAa data sets.

PL: Six QTLs were identified. QTLs *pl2b* and *pl5* were detected in the BCRIL, and the other four QTL pl2a, pl6, pl7, pl8 were detected only in GCA data sets. QTL *pl6* explained the phenotypic variation of 56.%.

FGPP: Two QTLs were detected. QTLs *fgpp1* was evaluated in the interval RM488-RM246 on chromosome 1, which was mapped in the BCRIL. The other QTL *fgpp3* was detected in the GCA data set.

SS: Four QTLs were resolved. Among them, QTL *ss3* and *ss11* were detected in the BCRIL and QTL *ss2* and *ss5* detected in GCA data sets, respectively.

GPP: Only one QTL *gpp6* was identified in the GCA data set. It explained for 58% of the observed variation.

SPP: Three QTLs were distributed on chromosomes 1, 4 or 6. Among them, two QTLs were identified in BCRIL (*spp1* and *spp4*), and one QTL *spp6* was found in the GCA data set.

GD: Three QTLs were found. QTLs *gd1* and *gd4* were identified in the BCRIL data set, and the other two QTLs, *gd1* and *gd3*, were found in the GCA data sets.

YD: Only one QTL was identified on chromosome 1 in the BCRIL data sets.

We found that most of the QTLs detected in Gca data set were also resolved in one, two or three Gca_i_ data sets in the same or in a nearby interval on the same chromosome (Table S5). Many of these QTLs might represent common QTLs because they demonstrated the same directionality and their LOD peaks appeared in a tightly linked genomic segment. This result further confirmed the feasibility of QTL mapping using a Gca data set.

Our results showed that the TC hybrid performance was related to the performance of the parental lines according to both the phenotypic correlation analysis and the QTL mapping. In BCRIL, 29 QTLs were identified. Among these QTLs, some QTLs that had a strong effect in the BCRILs (i.e., *ph8*, *hd7*, *hd8* and *tp4*) were also detected in identical or nearby intervals on the same chromosome in the Gca data set. In most instances of common main-effect QTLs across the BCRIL and Gca data sets, the direction of the parental contribution was identical. This result is consistent with the significant correlation coefficients observed between the BCRIL and Gca data sets for traits PH, HD and TP. When comparing the QTLs that were mapped in the BCRIL and Gca data sets, we considered that the Gca data set was likely to exhibit a partial proportion of the difference in performance due to any specific marker in comparison to the difference between the testers and the BCRILs. In the BCRILs, a QTL is identified when the additive effect between homozygous lines for the allele from the parents is significant, whereas the QTLs detected in the Gca data set are related to the additive effect of the BCRILs and the three testers.

### QTLs with a dominant effect

Table S6 shows the QTLs that were identified in the Sca and Hmp data sets in the three TC populations. In total, 93 main-effect QTLs were resolved in these six data sets. Consistent with the QTLs detected in the BCRIL and Gca data sets, most of these QTLs individually accounted for more than 10% of the observed variation.

PH: Four QTLs were distributed on chromosomes 4, 7 and 8. In the adjacent intervals (RM261-R4M30 and RM185-RM273) on chromosome 4, QTLs D-*ph4a* and D-*ph4b* were identified in Sca64s and Hmp64s, respectively. These two QTLs might represent the same QTL due to the same direction effect. QTL *D-ph7* and *D-ph8* were identified in Sca64s and Sca893s, respectively.

HD: Seven QTLs were distributed on chromosomes 2, 3, 4, 6, 8 and 9. *D*-*hd2*, *D*-*hd3* and *D*-*hd4a* were resolved in Sca888s. *D*-*hd4b* and *D*-*hd9* were detected in Sca64s and Sca893s, respectively. *D-hd6* was identified in Sca888s and Hmp888s. D-*hd8* was discovered simultaneously in Sca893s, Hmp888s and Hmp64s.

TP: Ten QTLs were mapped to chromosomes 2, 3, 4, 5 and 6. QTL D-*tp2*, D-*tp3a*, D-*tp3b*,D-*tp6a*, D-*tp6b* and *D-tp6c* were found in the different data sets. QTL D-*tp3a* revealed on chromosome 3 by MRG2180-RM6733 in Sca64s and D-*tp3b* by RM569-RM3392 in Sca893s were likely the same QTL (D-*tp3*) because the LOD- supported confidence intervals revealed a substantial overlap (map not shown). Similar cases were found in QTL D-*tp6a*, D-*tp6b* and *D-tp6c*. In addition, QTL D-*tp3c* was detected simultaneously in Sca893s and Sca888s with opposite direction effect.

PL: Seven QTLs were mapped on the chromosomes 1, 3, 4, 6, and 11. QTL D-*pl4* was detected simultaneously in Sca893s and Sca888s, with the opposite direction. And QTL D-*pl11* was identified simultaneously in Sca888s and Hmp888s, with the same direction. In addition, the LOD peaks for the QTLs *D-pl6a*, *D-pl6b* and *D-pl6c* in a tightly linked genomic segment of the same chromosome in the different TC population data sets suggested that they were the same QTL (D-*pl6*).

FGPP: Twelve QTLs were distributed throughout the genome, except chromosomes 9 and 10. In the adjacent intervals (RM151-RM8083 and RM6703-RM3362) on chromosome 1, QTLs *D-fgpp1a* and *D-fgpp1d* were identified in Sca893s and Sca888s, respectively. Some of the QTLs that were detected in nearby intervals on the same chromosome in the different TC population data sets might represent the same QTL based on their similar effects, such as *D-fgpp3*, *D-fgpp7*.

SS: Sixteen QTLs were distributed throughout the genome, except chromosomes 9 and 10. QTL D-*ss8* was identified simultaneously in Sca888s and Sca64s. Because of their similar effects, QTLs D-*ss4a* and D-*ss4b* might represent the same QTL, and they were detected in Sca888s and Sca64s, respectively. The QTLs D-*ss2a* and *D-ss2b* were identified on the same chromosome in the different TC population data sets, might represent the same QTL (*D-ss2*) because the confidence intervals substantially overlapped with a high LOD support (map not shown). Similar cases were observed for QTL *D-ss3*, *D-ss4*, *D-ss6* and *D-ss12*.

GPP: Seven QTLs were distributed on chromosomes 1, 2, 3, 6, 7, and 10. QTL *D-gpp3* was detected simultaneously in Hmp888s and Sca64s. Similarly, QTL D-*gpp6a* was identified simultaneously in Hmp888s and Hmp64s. Other individual QTL was revealed in only one data set.

SPP: Thirteen QTLs were distributed on chromosomes 1, 3, 4, 6, 7, 8, 11 and 12. With the opposite direction effects, QTL *D-spp1a* and *D-ssp1b* were detected in adjacent intervals on the same chromosome in Hmp893s and Sca888s data sets, respectively. Similar cases were observed between QTL *D-spp3a* and *D-ssp3b*, between *D*-*spp6a* and *D-spp6b*, and between *D-spp11a* and *D-ssp11b*. QTL *D-spp7b* was simultaneously detected in Sca893s and Sca64s data sets.

GD: Twelve QTLs were distributed on the chromosomes 1, 2, 3, 4, 9, 10, 11 and 12. QTLs D-*gd3a*, D-*gd3b* and D-*gd3c* identified on the same chromosome in the different TC population data sets might represent the same QTL (*D-gd3*) because the confidence intervals substantially overlapped with a high LOD support (map not shown). A similar phenomenon was observed for QTL *D-gd4* and *D-gd10*.

YD: Tree QTLs were detected on chromosomes 3, 7, and 11. QTL D-*yd11* was detected simultaneously in Sca888s and Hmp888s, with the same direction effects. The QTLs *D-yd3* and *D-yd7* were identified in Sca888s and Hmp893s data sets, respectively.

Most of QTLs with a dominant effect demonstrated the ability to individually explain more than 10% of the phenotypic variation in this study. However, the data obtained in other QTL analyses performed in rice [21], [27]–[29] showed the variation of individual QTL was less than 10%. Which result is more reasonable or correct remains to be further validated. This difference might be due to the application of different materials and procedures among those studies. Interestingly, some QTLs that were detected in Sca were also mapped in the same or in adjacent intervals of the same chromosome in Hmp for identical TC populations. Many of these QTLs might represent the same QTL because a substantial overlap of the LOD support confidence intervals was observed (data not shown). In addition, the effects of the common main-effect QTLs between the Sca and Hmp data sets may vary with respect to the magnitude of their substitution effects; however, the parental contribution does not change. This phenomenon is consistent with the significant correlation of all of the evaluated traits between the Sca and Hmp data sets. Some common QTLs that were also detected in the Sca or Hmp data sets in different TC populations affected the same trait, such as D-*hd8* and D-*tp3c*, among others. However, the direction of these QTLs in different TC populations was the same in some cases, and different in the other cases. This finding is in contrast to the results of some empirical studies of other self-pollinating and out-crossing plant species [21]–[22], [30]. This discrepancy may be explained by the multiple alleles that are present at certain loci in the three testers, which resulted in a mixture of dominant and epistatic effects of the QTLs in the Sca and Hmp data sets.

Although a large number of loci related to heterosis have been identified in rice, there are limited successful applications of these loci using MAS (marker assisted selection; [5]). Yu *et al*. [31] have suggested that this limitation might be due to several phenomena. First, the expression of a QTL that is related to heterosis is influenced by the genetic background. In other words, the heterosis-related loci were identified in some combinations but could not be detected in others due to the different genetic backgrounds. Second, heterosis loci were always detected based on a pair of special alleles; if the alleles changed, then the magnitude of the heterosis effect and even its direction might differ.

### Cluster distribution of the main-effect QTL

An interesting result of the present study is the highly concentrated distribution of QTLs in a few chromosomal regions and the presence of QTL hot spots (Tables S6 and S7, Fig. 2). These findings are particularly true for the region surrounding the RM462-RM1247-RM6324 locus on chromosome 1, the RM589-RM314-RM50-RM121 locus on chromosome 6, and the RM1253-RM3583 locus on chromosome 7, the RM25-MRG2181 locus on chromosome 8, where the QTLs for several traits were detected in the BCRIL, Gca, Sca and Hmp data sets in the three TC populations. Similar concentrated distributions of QTLs have also been observed in previous studies [30], [32]. Particular attention should be given to such QTL hot spots in future studies for gene cloning and functional genomics.

In conclusion, combining ability and heterosis of agronomic traits could be analyzed by QTL mapping method. The characteristics of the QTLs for combining ability of agronomic traits were similar with those for agronomic traits with performance *per se* of BCRIL. Several QTLs (1∼6 in this study) were identified for each trait for combining ability. Some QTLs were pleiotropic or tightly linked with each other. The identification of QTLs responsible for combining ability and heterosis in the present study provides valuable information for dissecting genetic basis of combining ability and heterosis.

## Supporting Information

Table S1 **The genotype and genotype effect of marker and QTL for combining ability and heterosis with two alleles at each locus in RIL/DH population.**
(DOC)

Table S2 **The mean value of RIL/DH population, TC populations, Hmp, Sca and Gca data sets with two alleles at each locus in RIL/DH population.**
(DOC)

Table S3 **The genotype and genotype effect of marker and QTL for combining ability and heterosis with multiple alleles at each locus in RIL/DH population.**
(DOC)

Table S4 **The mean value of RIL/DH population, TC populations, Hmp, Sca and Gca data sets with multiple alleles at each locus in RIL/DH population.**
(DOC)

Table S5 **QTL detected in BCRIL and Gca and Gcai data set.**
(DOC)

Table S6 Main-effect QTL detected in BCRIL and Gca data sets.
(DOC)

Table S7 Main-effect QTL detected in Sca and Hmp data sets of three TC populations.
(DOC)
