# Correction: QTL Mapping of Combining Ability and Heterosis of Agronomic Traits in Rice Backcross Recombinant Inbred Lines and Hybrid Crosses

**DOI:** 10.1371/annotation/f7108c9e-bab9-421f-9c0b-1227a421399b

**Published:** 2012-03-08

**Authors:** Zhen Qu, Lanzhi Li, Junyuan Luo, Peng Wang, Sibin Yu, Tongmin Mou, Xingfei Zheng, Zhongli Hu

There was an error in affiliations 2 and 3. The affiliations were reversed. The correct affiliations are:

2 Hunan Provincial Key Laboratory for Biology and Control of Plant Disease and Insect Pests, College of Bio-Safety Science and Technology, Hunan Agricultural University, Changsha, China,

3 Key Laboratory of Oil Crop Biology of the Ministry of Agriculture, Oil Crops Research Institute, Chinese Academy of Agricultural Sciences, Wuhan, China

