# Correction: QTL Mapping of Combining Ability and Heterosis of Agronomic Traits in Rice Backcross Recombinant Inbred Lines and Hybrid Crosses

**DOI:** 10.1371/annotation/df08f19f-7538-4726-ba69-1ba41370732a

**Published:** 2012-06-13

**Authors:** Zhen Qu, Lanzhi Li, Junyuan Luo, Peng Wang, Sibin Yu, Tongmin Mou, Xingfei Zheng, Zhongli Hu

There is an additional error in the affiliations. The correct affiliations are:

Zhen Qu1,Lanzhi Li1,2, Junyuan Luo3, Peng Wang3, Sibin Yu3, Tongmin Mou3*, Xingfei Zheng1,Zhongli Hu1*

1 State Key Laboratory of Hybrid Rice, College of Life Science, Wuhan University, Wuhan, China,

2 Hunan Provincial Key Laboratory for Biology and Control of Plant Disease and Insect Pests, College of Bio-Safety Science and Technology, Hunan Agricultural University, Changsha, China,

3 National Key Laboratory of Crop Genetic Improvement, Huazhong Agricultural University, Wuhan, China.

