# Supplementary material for: QTL Mapping of Combining Ability and Heterosis of Agronomic Traits in Rice Backcross Recombinant Inbred Lines and Hybrid Crosses
Source: PLoS One. 2012 Jan 26;7(1):e28463. doi: 10.1371/journal.pone.0028463 (PMC3266898; doi:10.1371/journal.pone.0028463)
Supplement: Table S1 — The genotype and genotype effect of marker and QTL for combining ability and heterosis with two alleles at each locus in RIL/DH population. (DOC) [file pone.0028463.s001.doc]

Table S1 The genotype and genotype effect of marker and QTL for combining ability and heterosis with two alleles at each locus in DH population

|  |  | *MM* | |  | *mm* | |
| --- | --- | --- | --- | --- | --- | --- |
| Genotype in DH population |  | *MMQQ* | *MMqq* |  | *mmQQ* | *mmqq* |
| Genotype effect in DH population |  | $\mu+a$ | $\mu-a$ |  | $\mu+a$ | $\mu-a$ |
| Genotype frequency in DH population |  | $\frac{1}{2}(1-r)$ | $\frac{1}{2}r$ |  | $\frac{1}{2}r$ | $\frac{1}{2}(1-r)$ |
|  |  |  |  |  |  |  |
| *Genotype in TC population |  | *MMQQ* | *MMQq* |  | *mmQQ* | *mmQq* |
| *Genotype effect in TC population |  | $\mu+a$ | $\mu+d$ |  | $\mu+a$ | $\mu+d$ |
| *Genotype frequency in TC population |  | $\frac{1}{2}(1-r)$ | $\frac{1}{2}r$ |  | $\frac{1}{2}r$ | $\frac{1}{2}(1-r)$ |
| *Genotype effect in Hmp data set |  | $0$ | $d$ |  | $0$ | $d$ |
| *Genotype effect in Sca data set |  | $-qd$ | $qd$ |  | $-qd$ | $qd$ |
|  |  |  |  |  |  |  |
| #Genotype in TC population |  | *MMQq* | *MMqq* |  | *mmQq* | *mmqq* |
| #Genotype effect in TC population |  | $\mu+d$ | $\mu-a$ |  | $\mu+d$ | $\mu-a$ |
| #Genotype frequency in TC population |  | $\frac{1}{2}(1-r)$ | $\frac{1}{2}r$ |  | $\frac{1}{2}r$ | $\frac{1}{2}(1-r)$ |
| #Genotype effect in Hmp data set |  | $d$ | $0$ |  | $d$ | $0$ |
| #Genotype effect in Sca data set |  | $pd$ | $-pd$ |  | $pd$ | $-pd$ |
|  |  |  |  |  |  |  |
| §Genotype effect in TC population |  | $\mu+pa+qd$ | $\mu-qa+pd$ |  | $\mu+pa+qd$ | $\mu-qa+pd$ |
| §Genotype effect in Gca data set |  | $\frac{1}{2}a+(q-\frac{1}{2})d$ | $-\frac{1}{2}a-(q-\frac{1}{2})d$ |  | $\frac{1}{2}a+(q-\frac{1}{2})d$ | $-\frac{1}{2}a-(q-\frac{1}{2})d$ |

*MM* and *mm* denote the two genotype of molecular marker M; *QQ*, *Qq* and *qq* denote the three genotype of QTL; *r* represents the recombinant probability between molecular marker M and QTL in DH population;*μ*denote the overall mean value. *a* and *d* denote the additive effect and dominant effect, respectively; *q* and *p* denote the genotype frequency of QTL *QQ* and *qq* in tester, respectively (*p+q*=1).

For the RI population, the expectations were similar to those in the DH population except for r, which was replaced by and , respectively. The and were recombinant values for two RI populations (selﬁng population and sib-mating population), respectively(Hu et al. 2002).

*When the genotype of QTL in tester is *QQ*,*,* and its genotype frequency is *q*

# When the genotype of QTL in tester is *qq*,*,* and its genotype frequency is *p*

§When the genotype of QTL in tester is a mixture of *QQ* and *qq*, the genotype frequency of *QQ* and *qq* are *q* and *p*, respectively.
