# Supplementary material for: QTL Mapping of Combining Ability and Heterosis of Agronomic Traits in Rice Backcross Recombinant Inbred Lines and Hybrid Crosses
Source: PLoS One. 2012 Jan 26;7(1):e28463. doi: 10.1371/journal.pone.0028463 (PMC3266898; doi:10.1371/journal.pone.0028463)
Supplement: Table S2 — The mean value of RIL/DH population, TC populations, Hmp, Sca and Gca data sets with two alleles at each locus in RIL/DH population. (DOC) [file pone.0028463.s002.doc]

Table S2 The mean value of DH population, TC populations, Hmp, Sca and Gca data sets with two alleles at each locus in DH population

| Data set |  | Mean value |
| --- | --- | --- |
| DH population |  | $\mu$ |
| * TC population |  | $\mu+\frac{1}{2}(a+d)$ |
| * Hmp data set |  | $-\frac{1}{2}d(1-2r)$ |
| * Sca data set |  | $-qd(1-2r)$ |
| # TC population |  | $\mu+\frac{1}{2}(-a+d)$ |
| # Hmp data set |  | $\frac{1}{2}d(1-2r)$ |
| # Sca data set |  | $pd(1-2r)$ |
| §TC population |  | $\mu+\frac{1}{2}(p-q)a+\frac{1}{2}d$ |
| §Gca data set |  | $[\frac{1}{2}a+(q-\frac{1}{2})d](1-2r)$ |

*MM* and *mm* denote the two genotype of molecular marker M; *QQ*, *Qq* and *qq* denote the three genotype of QTL; *r* represents the recombinant probability between molecular marker M and QTL in DH population;*μ*denote the overall mean value. *a* and *d* denote the additive effect and dominant effect, respectively; *q* and *p* denote the genotype frequency of QTL *QQ* and *qq* in tester, respectively (*p+q*=1).

For the RI population, the expectations were similar to those in the DH population except for r, which was replaced by and , respectively. The and were recombinant values for two RI populations (selﬁng population and sib-mating population), respectively(Hu et al. 2002).

*When the genotype of QTL in tester is *QQ*,*,* and its genotype frequency is *q*

# When the genotype of QTL in tester is *qq*,*,* and its genotype frequency is *p*

§When the genotype of QTL in tester is a mixture of *QQ* and *qq*, the genotype frequency of *QQ* and *qq* are *q* and *p*, respectively.
