# Supplementary material for: QTL Mapping of Combining Ability and Heterosis of Agronomic Traits in Rice Backcross Recombinant Inbred Lines and Hybrid Crosses
Source: PLoS One. 2012 Jan 26;7(1):e28463. doi: 10.1371/journal.pone.0028463 (PMC3266898; doi:10.1371/journal.pone.0028463)
Supplement: Table S3 — The genotype and genotype effect of marker and QTL for combining ability and heterosis with multiple alleles at each locus in RIL/DH population. (DOC) [file pone.0028463.s003.doc]

Table S3 The genotype and genotype effect of marker and QTL for combining ability and heterosis with multiple alleles at each locus in DH population

|  |  | *MM* | |  | *mm* | |
| --- | --- | --- | --- | --- | --- | --- |
| Genotype in DH population |  | *MMQQ* | *MMqq* |  | *mmQQ* | *mmqq* |
| Genotype effect in DH population |  | $\mu+a$ | $\mu-a$ |  | $\mu+a$ | $\mu-a$ |
| Genotype frequency in DH population |  | $\frac{1}{2}(1-r)$ | $\frac{1}{2}r$ |  | $\frac{1}{2}r$ | $\frac{1}{2}(1-r)$ |
|  |  |  |  |  |  |  |
| *Genotype in TC population |  | $MMQ_{1}Q$ | $MMQ_{1}q$ |  | $mmQ_{1}Q$ | $mmQ_{1}q$ |
| *Genotype effect in TC population |  | $\mu+g_{1}$ | $\mu+g_{1}^{'}$ |  | $\mu+g_{1}$ | $\mu+g_{1}^{'}$ |
| *Genotype frequency in TC population |  | $\frac{1}{2}(1-r)$ | $\frac{1}{2}r$ |  | $\frac{1}{2}r$ | $\frac{1}{2}(1-r)$ |
| *Genotype effect in Hmp data set |  | $g_{1}-\frac{1}{2}(2\mu+a_{1}+a)$ | $g_{1}^{'}-\frac{1}{2}(2\mu+a_{1}-a)$ |  | $g_{1}-\frac{1}{2}(2\mu+a_{1}+a)$ | $g_{1}^{'}-\frac{1}{2}(2\mu+a_{1}-a)$ |
| *Genotype effect in Sca data set |  | $\frac{1}{2}(g_{1}-g_{1}^{'})-\frac{1}{2}(G-G')$ | $-\frac{1}{2}(g_{1}-g_{1}^{'})+\frac{1}{2}(G-G')$ |  | $\frac{1}{2}(g_{1}-g_{1}^{'})-\frac{1}{2}(G-G')$ | $-\frac{1}{2}(g_{1}-g_{1}^{'})+\frac{1}{2}(G-G')$ |
| … |  | … | … |  | … | … |
| #Genotype in TC population |  | $MMQ_{k}Q$ | $MMQ_{k}q$ |  | $mmQ_{k}Q$ | $mmQ_{k}q$ |
| #Genotype effect in TC population |  | $\mu+d$ | $\mu-a$ |  | $\mu+d$ | $\mu-a$ |
| #Genotype frequency in TC population |  | $\frac{1}{2}(1-r)$ | $\frac{1}{2}r$ |  | $\frac{1}{2}r$ | $\frac{1}{2}(1-r)$ |
| #Genotype effect in Hmp data set |  | $g_{k}-\frac{1}{2}(2\mu+a_{k}+a)$ | $g_{k}^{'}-\frac{1}{2}(2\mu+a_{k}-a)$ |  | $g_{k}-\frac{1}{2}(2\mu+a_{k}+a)$ | $g_{k}^{'}-\frac{1}{2}(2\mu+a_{k}-a)$ |
| #Genotype effect in Sca data set |  | $\frac{1}{2}(g_{k}-g_{k}^{'})-\frac{1}{2}(G-G')$ | $-\frac{1}{2}(g_{k}-g_{k}^{'})+\frac{1}{2}(G-G')$ |  | $\frac{1}{2}(g_{k}-g_{k}^{'})-\frac{1}{2}(G-G')$ | $-\frac{1}{2}(g_{k}-g_{k}^{'})+\frac{1}{2}(G-G')$ |
| #Genotype effect in Gca data set |  | $\frac{1}{2}(G-G')$ | $-\frac{1}{2}(G-G')$ |  | $\frac{1}{2}(G-G')$ | $-\frac{1}{2}(G-G')$ |

*MM* and *mm* denote the two different genotype of molecular marker M; *Q* and *q* denote two alleles of QTL in DH population, Q_i_ (i=1~k) represents the multiple alleles of QTL in tester; *r* represents the recombinant value between molecular marker M and QTL in DH population; *μ*idenotes the overall mean value. *a*, *a*_1_ and *a*_k_ denote the additive effect of different allele; *g_i_* and *g_i_’* (1~k) denote the genotypic value of the homozygote and heterozygote of QTL, respectively. $G=\frac{1}{k}\sum_{i=1}^{k} g_{i}$，$G'=\frac{1}{k}\sum_{i=1}^{k} g'_{i}$. For the RI population, the expectations were similar to those in the DH population except for r, which was replaced by and , respectively. The and were recombinant values for two RI populations (selﬁng population and sib-mating population), respectively(Hu et al. 2002).

*When the genotype of QTL is *Q*_1_*Q*_1_ in tester

# When the genotype of QTL is *Q*_k_*Q*_k_ in tester
