# Supplementary material for: QTL Mapping of Combining Ability and Heterosis of Agronomic Traits in Rice Backcross Recombinant Inbred Lines and Hybrid Crosses
Source: PLoS One. 2012 Jan 26;7(1):e28463. doi: 10.1371/journal.pone.0028463 (PMC3266898; doi:10.1371/journal.pone.0028463)
Supplement: Table S4 — The mean value of RIL/DH population, TC populations, Hmp, Sca and Gca data sets with multiple alleles at each locus in RIL/DH population. (DOC) [file pone.0028463.s004.doc]

Table S4 The mean value of DH population, TC populations, Hmp, Sca and Gca data sets with multiple alleles at each locus in DH population

| Data set |  | Mean value |
| --- | --- | --- |
| DH population |  | $\mu$ |
| * TC population |  | $\mu+\frac{1}{2}(g_{1}+g_{1}^{'})$ |
| * Hmp data set |  | $\frac{1}{2}(g_{1}-g_{1}^{'}-a)(1-2r)$ |
| * Sca data set |  | $\frac{1}{2}[(g_{1}-g_{1}')-(G-G')](1-2r)$ |
| # TC population |  | $\mu+\frac{1}{2}(g_{k}+g_{k}^{'})$ |
| # Hmp data set |  | $\frac{1}{2}(g_{k}-g_{k}^{'}-a)(1-2r)$ |
| # Sca data set |  | $\frac{1}{2}[(g_{k}-g_{k}')-(G-G')](1-2r)$ |
| # Gca data set |  | $\frac{1}{2}(G-G')(1-2r)$ |

*MM* and *mm* denote the two different genotype of molecular marker M; *Q* and *q* denote two alleles of QTL in DH population, Q_i_ (i=1~k) represents the multiple alleles of QTL in tester; *r* represents the recombinant value between molecular marker M and QTL in DH population; *μ*idenotes the overall mean value. *a*, *a*_1_ and *a*_k_ denote the additive effect of different allele; *g_i_* and *g_i_’* (1~k) denote the genotypic value of the homozygote and heterozygote of QTL, respectively. $G=\frac{1}{k}\sum_{i=1}^{k} g_{i}$，$G'=\frac{1}{k}\sum_{i=1}^{k} g'_{i}$. For the RI population, the expectations were similar to those in the DH population except for r, which was replaced by and , respectively. The and were recombinant values for two RI populations (selﬁng population and sib-mating population), respectively(Hu et al. 2002).

*When the genotype of QTL is *Q*_1_*Q*_1_ in tester

# When the genotype of QTL is *Q*_k_*Q*_k_ in tester
