# Supplementary material for: QTL Mapping of Combining Ability and Heterosis of Agronomic Traits in Rice Backcross Recombinant Inbred Lines and Hybrid Crosses
Source: PLoS One. 2012 Jan 26;7(1):e28463. doi: 10.1371/journal.pone.0028463 (PMC3266898; doi:10.1371/journal.pone.0028463)
Supplement: Table S5 — QTL detected in BCRIL and Gca and Gcai data set. (DOC) [file pone.0028463.s005.doc]

Table S5 QTL detected in BCRIL and Gca and Gcai data set

|  |  |  |  | BCRIL | | | Gca | | | Gca_1_^b^ | | | Gca_2_^b^ | | | Gca_3_^b^ | | |
| --- | --- | --- | --- | --- | --- | --- | --- | --- | --- | --- | --- | --- | --- | --- | --- | --- | --- | --- |
| Trait | QTL | Chr^a^ | Interval | LOD | A | *R*^2^(%) | LOD | A | *R*^2^(%) | LOD | A | *R*^2^(%) | LOD | A | *R*^2^(%) | LOD | A | *R*^2^(%) |
| PH | *ph3a* | 3 | RM532-RM520 |  |  |  | 4.06 | -3.16 | 24.04 | 3.75 | -3.24 | 22.29 | 3.64 | -3.31 | 26.87 |  |  |  |
| PH | *ph3b* | 3 | RM520-RM293 |  |  |  |  |  |  |  |  |  |  |  |  | 3.11 | -2.64 | 11.23 |
| PH | *ph4a* | 4 | RM261-R4M30 |  |  |  |  |  |  | 2.54 | -1.83 | 10.38 |  |  |  |  |  |  |
| PH | *ph4b* | 4 | RM185-RM273 |  |  |  | 2.19 | -1.65 | 6.90 |  |  |  |  |  |  | 2.58 | -1.97 | 7.74 |
| PH | *ph4c* | 4 | RM273-RM252 | 3.86 | 3.68 | 16.91 |  |  |  |  |  |  |  |  |  |  |  |  |
| PH | *ph5a* | 5 | RM440-RM3575 |  |  |  |  |  |  | 3.05 | -2.30 | 11.34 |  |  |  |  |  |  |
| PH | *ph5b* | 5 | RM3321-RM480 |  |  |  |  |  |  |  |  |  | 2.11 | -2.07 | 7.75 |  |  |  |
| PH | *ph7* | 7 | RM3583-RM7110 |  |  |  | 5.45 | -3.49 | 25.19 | 9.24 | -4.54 | 38.47 |  |  |  | 3.99 | -3.41 | 18.89 |
| PH | *ph8* | 8 | RM25-MRG2181 | 4.77 | -5.36 | 21.31 | 4.51 | -3.55 | 25.53 | 3.75 | -3.44 | 21.15 |  |  |  | 5.71 | -4.38 | 30.20 |
| PH | *ph12a* | 12 | RM3717-RM19 |  |  |  | 3.07 | -1.84 | 11.25 |  |  |  |  |  |  |  |  |  |
| PH | *ph12b* | 12 | RM19-RM3472 |  |  |  |  |  |  |  |  |  | 4.66 | -2.49 | 21.38 | 2.47 | -1.80 | 8.46 |
|  |  |  |  |  |  |  |  |  |  |  |  |  |  |  |  |  |  |  |
| HD | *hd1* | 1 | RM151-RM8083 | 2.83 | -1.12 | 7.99 |  |  |  |  |  |  |  |  |  |  |  |  |
| HD | *hd2* | 2 | RM526-RM530 |  |  |  |  |  |  |  |  |  | 3.24 | 1.72 | 16.75 |  |  |  |
| HD | *hd3* | 3 | RM520-RM293 | 4.39 | -2.01 | 12.95 |  |  |  |  |  |  |  |  |  |  |  |  |
| HD | *hd6* | 6 | RM584-RM314 | 5.43 | 3.17 | 17.37 |  |  |  |  |  |  |  |  |  |  |  |  |
| HD | *hd7* | 7 | RM1253-RM3583 | 9.89 | -2.98 | 36.92 | 4.07 | -2.83 | 24.18 | 5.17 | -3.68 | 30.28 | 3.12 | -2.27 | 20.81 |  |  |  |
| HD | *hd8* | 8 | RM25-MRG2181 | 5.45 | -2.70 | 22.80 | 4.87 | -3.24 | 21.65 | 4.32 | -3.38 | 18.54 |  |  |  | 5.62 | -4.25 | 27.77 |
|  |  |  |  |  |  |  |  |  |  |  |  |  |  |  |  |  |  |  |
| TP | *tp1a* | 1 | RM462-RM1247 | 3.03 | 0.51 | 18.78 |  |  |  |  |  |  |  |  |  |  |  |  |
| TP | *tp1b* | 1 | RM151-RM8083 |  |  |  | 3.07 | 1.36 | 33.40 |  |  |  |  |  |  |  |  |  |
| TP | *tp2a* | 2 | RM5862-RM7355 |  |  |  | 2.93 | 1.69 | 28.33 |  |  |  | 2.10 | 1.29 | 19.92 |  |  |  |
| TP | *tp2b* | 2 | RM561-RM6318 |  |  |  |  |  |  | 2.76 | -2.25 | 29.77 |  |  |  |  |  |  |
| TP | *tp2c* | 2 | RM6318-RM526 | 2.47 | -0.49 | 12.94 |  |  |  |  |  |  |  |  |  |  |  |  |
| TP | *tp4a* | 4 | RM307-RM261 | 3.42 | 0.72 | 21.28 |  |  |  |  |  |  |  |  |  |  |  |  |
| TP | *tp4b* | 4 | RM261-R4M30 |  |  |  | 2.74 | 1.39 | 25.89 |  |  |  |  |  |  | 2.74 | 2.12 | 31.58 |
| TP | *tp5* | 5 | RM3321-RM480 | 5.52 | 0.86 | 35.69 |  |  |  |  |  |  |  |  |  |  |  |  |
| TP | *tp6a* | 6 | RM589-RM584 |  |  |  | 3.17 | -1.66 | 23.97 |  |  |  | 4.03 | -3.71 | 29.07 |  |  |  |
| TP | *tp6b* | 6 | RM584-RM314 |  |  |  |  |  |  |  |  |  |  |  |  | 2.09 | 1.56 | 25.53 |
| TP | *tp6c* | 6 | RM121-RM6071 |  |  |  | 3.07 | 2.18 | 35.09 |  |  |  |  |  |  |  |  |  |
| TP | *tp7* | 7 | RM3583-RM7110 |  |  |  |  |  |  | 2.02 | 1.17 | 11.93 | 2.90 | 1.52 | 18.82 |  |  |  |
| TP | *tp11* | 11 | RM6327-RM1812 | 2.33 | -0.64 | 21.59 |  |  |  |  |  |  |  |  |  |  |  |  |
| TP | *tp12* | 12 | MRG0986-RM3331 |  |  |  | 2.21 | 0.87 | 14.93 |  |  |  |  |  |  |  |  |  |
|  |  |  |  |  |  |  |  |  |  |  |  |  |  |  |  |  |  |  |
| PL | *pl1* | 1 | RM462-RM1247 | 2.54 | 10.97 | 15.00 |  |  |  |  |  |  |  |  |  |  |  |  |
| PL | *pl2a* | 2 | RM5862-RM7355 |  |  |  | 3.03 | 47.80 | 23.45 |  |  |  | 2.20 | 44.06 | 21.33 |  |  |  |
| PL | *pl2b* | 2 | RM6318-RM526 | 3.77 | -15.60 | 22.92 |  |  |  |  |  |  |  |  |  |  |  |  |
| PL | *pl3a* | 3 | RM569-RM3392 |  |  |  | 2.41 | 47.88 | 23.47 |  |  |  |  |  |  |  |  |  |
| PL | *pl3b* | 3 | MRG5959-MRG2180 | 2.05 | 12.72 | 15.18 |  |  |  |  |  |  |  |  |  |  |  |  |
| PL | *pl4* | 4 | RM261-R4M30 |  |  |  |  |  |  |  |  |  | 2.81 | 34.42 | 19.85 |  |  |  |
| PL | *pl5* | 5 | RM3295-RM7081 | 3.54 | 16.91 | 27.58 |  |  |  |  |  |  |  |  |  |  |  |  |
| PL | *pl6a* | 6 | RM314-RM50 |  |  |  | 4.27 | 54.15 | 33.06 | 6.84 | 63.08 | 41.32 | 3.41 | 50.87 | 29.02 |  |  |  |
| PL | *pl6b* | 6 | RM121-RM6071 |  |  |  | 5.55 | 51.29 | 56.17 |  |  |  |  |  |  | 4.18 | 79.12 | 58.81 |
| PL | *pl7* | 7 | RM3583-RM7110 |  |  |  | 4.02 | 42.26 | 21.45 |  |  |  |  |  |  |  |  |  |
| PL | *pl8a* | 8 | RM506-RM152 |  |  |  |  |  |  | 2.72 | 16.24 | 12.29 |  |  |  |  |  |  |
| PL | *pl8b* | 8 | RM152-MRG0270 |  |  |  | 3.14 | 51.20 | 31.05 |  |  |  |  |  |  |  |  |  |
|  |  |  |  |  |  |  |  |  |  |  |  |  |  |  |  |  |  |  |
| FGPP | *fgpp1a* | 1 | RM488-RM246 | 4.45 | -151.01 | 41.14 |  |  |  |  |  |  |  |  |  |  |  |  |
| FGPP | *fgpp1b* | 1 | RM6666-RM212 |  |  |  | 2.19 | -140.04 | 11.68 |  |  |  | 3.14 | -196.27 | 18.94 |  |  |  |
| FGPP | *fgpp3* | 3 | MRG5959-MRG2180 | |  |  | 3.62 | 203.69 | 23.80 |  |  |  | 4.38 | 224.49 | 25.94 | 4.10 | 291.66 | 30.78 |
| FGPP | *fgpp4* | 4 | RM252-RM241 |  |  |  | 2.16 | -94.40 | 9.22 |  |  |  | 2.61 | -99.02 | 9.45 |  |  |  |
| FGPP | *fgpp5* | 5 | RM7081-RM3321 | 2.16 | 101.59 | 14.39 |  |  |  |  |  |  |  |  |  |  |  |  |
| FGPP | *fgpp6a* | 6 | RM584-RM314 |  |  |  | 2.80 | 216.72 | 24.88 |  |  |  |  |  |  |  |  |  |
| FGPP | *fgpp6b* | 6 | RM314-RM50 |  |  |  |  |  |  | 3.36 | 173.32 | 15.42 |  |  |  |  |  |  |
| FGPP | *fgpp6c* | 6 | RM121-RM6071 |  |  |  |  |  |  |  |  |  |  |  |  | 3.78 | 500.91 | 40.28 |
| FGPP | *fgpp7a* | 7 | RM51-RM3325 |  |  |  | 2.97 | 202.00 | 26.30 |  |  |  | 4.74 | 230.97 | 32.21 |  |  |  |
| FGPP | *fgpp7b* | 7 | RM3325-RM1253 |  |  |  |  |  |  |  |  |  |  |  |  | 3.22 | 269.82 | 26.09 |
| FGPP | *fgpp8* | 8 | RM506-RM152 |  |  |  |  |  |  | 2.59 | 104.08 | 11.25 |  |  |  |  |  |  |
|  |  |  |  |  |  |  |  |  |  |  |  |  |  |  |  |  |  |  |
| SS | *ss2a* | 2 | RM5862-RM7355 | 2.37 | 4.41 | 12.06 |  |  |  |  |  |  |  |  |  |  |  |  |
| SS | *ss2b* | 2 | RM5699-RM324 |  |  |  | 3.96 | -3.79 | 25.09 |  |  |  |  |  |  | 3.14 | -3.87 | 20.61 |
| SS | *ss2c* | 2 | RM561-RM6318 |  |  |  |  |  |  | 3.89 | -4.67 | 26.86 |  |  |  |  |  |  |
| SS | *ss2d* | 2 | RM530-RM5916 |  |  |  | 2.28 | 2.84 | 17.14 |  |  |  |  |  |  |  |  |  |
| SS | *ss3a* | 3 | RM3392-RM5925 | 5.90 | -5.73 | 36.11 |  |  |  |  |  |  |  |  |  |  |  |  |
| SS | *ss3b* | 3 | MRG4626-R3M37 |  |  |  |  |  |  | 2.89 | 3.97 | 17.91 |  |  |  |  |  |  |
| SS | *ss3c* | 3 | R3M37-RM532 |  |  |  |  |  |  |  |  |  | 3.19 | 3.51 | 16.76 |  |  |  |
| SS | *ss5a* | 5 | RM440-RM3575 |  |  |  | 3.55 | 3.25 | 27.53 |  |  |  | 5.27 | 4.98 | 36.33 | 2.42 | 2.88 | 16.64 |
| SS | *ss5b* | 5 | RM3321-RM480 |  |  |  |  |  |  |  |  |  | 3.92 | -4.12 | 26.55 |  |  |  |
| SS | *ss7* | 7 | RM473A-MRG2555 |  |  |  | 2.19 | -2.22 | 12.44 |  |  |  |  |  |  |  |  |  |
| SS | *ss8* | 8 | RM483-RM342 |  |  |  |  |  |  | 3.16 | -4.28 | 33.17 |  |  |  |  |  |  |
| SS | *ss9* | 9 | RM553-RM215 | 2.10 | -3.63 | 15.58 |  |  |  |  |  |  |  |  |  |  |  |  |
| SS | *ss10* | 10 | MRG6300-RM228 |  |  |  |  |  |  |  |  |  |  |  |  | 2.13 | 3.04 | 14.93 |
| SS | *ss11* | 11 | RM1812-MRG5615 | 4.49 | -4.44 | 30.44 |  |  |  | 2.50 | -2.97 | 24.67 |  |  |  |  |  |  |
|  |  |  |  |  |  |  |  |  |  |  |  |  |  |  |  |  |  |  |
| GPP | *gpp6a* | 6 | RM314-RM50 |  |  |  |  |  |  | 5.00 | 508.55 | 34.29 |  |  |  |  |  |  |
| GPP | *gpp6b* | 6 | RM121-RM6071 |  |  |  | 4.08 | 954.03 | 58.73 |  |  |  |  |  |  | 6.64 | 774.77 | 65.68 |
| GPP | *gpp7a* | 7 | RM51-RM3325 |  |  |  |  |  |  |  |  |  | 3.34 | 341.90 | 33.09 |  |  |  |
| GPP | *gpp7b* | 7 | RM3325-RM1253 |  |  |  |  |  |  |  |  |  |  |  |  | 4.49 | 677.57 | 26.52 |
| GPP | *gpp7c* | 7 | RM3583-RM7110 |  |  |  | 2.16 | 323.63 | 14.83 |  |  |  |  |  |  |  |  |  |
| GPP | *gpp10* | 10 | MRG4348-RM5689 |  |  |  |  |  |  |  |  |  | 2.56 | -205.98 | 14.55 |  |  |  |
|  |  |  |  |  |  |  |  |  |  |  |  |  |  |  |  |  |  |  |
| SPP | *spp1a* | 1 | RM462-RM1247 | 3.23 | -8.49 | 13.62 |  |  |  |  |  |  |  |  |  |  |  |  |
| SPP | *spp1b* | 1 | RM283-RM151 |  |  |  |  |  |  |  |  |  | 2.01 | -7.94 | 10.19 |  |  |  |
| SPP | *spp1c* | 1 | RM212-RM6703 | 2.44 | 6.56 | 7.09 |  |  |  |  |  |  |  |  |  |  |  |  |
| SPP | *spp2a* | 2 | RM5862-RM7355 |  |  |  |  |  |  |  |  |  | 2.17 | -10.19 | 15.32 |  |  |  |
| SPP | *spp2b* | 2 | RM7355-RM5699 |  |  |  | 2.31 | -11.01 | 14.76 |  |  |  |  |  |  |  |  |  |
| SPP | *spp2c* | 2 | RM561-RM6318 |  |  |  |  |  |  | 2.47 | 17.98 | 41.81 |  |  |  |  |  |  |
| SPP | *spp3* | 3 | MRG5959-MRG2180 | |  |  |  |  |  |  |  |  |  |  |  | 3.18 | 14.90 | 34.26 |
| SPP | *spp4* | 4 | RM252-RM241 | 4.53 | 9.32 | 19.40 |  |  |  |  |  |  |  |  |  |  |  |  |
| SPP | *spp6a* | 6 | RM584-RM314 |  |  |  |  |  |  | 2.66 | 22.69 | 17.66 | 3.24 | 16.37 | 22.16 |  |  |  |
| SPP | *spp6b* | 6 | RM314-RM50 |  |  |  | 3.43 | 12.98 | 17.84 |  |  |  |  |  |  | 4.88 | 19.76 | 32.23 |
| SPP | *spp7a* | 7 | RM3583-RM7110 |  |  |  |  |  |  |  |  |  |  |  |  | 3.13 | -15.36 | 17.66 |
| SPP | *spp7b* | 7 | RM3753-RM473A |  |  |  |  |  |  |  |  |  | 2.01 | 6.03 | 7.15 |  |  |  |
| SPP | *spp11* | 11 | RM202-RM287 | 2.56 | -8.77 | 11.01 |  |  |  |  |  |  |  |  |  |  |  |  |
|  |  |  |  |  |  |  |  |  |  |  |  |  |  |  |  |  |  |  |
| GD | *gd1a* | 1 | RM462-RM1247 | 2.34 | -0.36 | 14.19 |  |  |  |  |  |  |  |  |  |  |  |  |
| GD | *gd1b* | 1 | RM283-RM151 |  |  |  | 3.66 | -0.46 | 25.00 | 2.36 | -0.43 | 17.56 | 4.22 | -0.50 | 27.35 |  |  |  |
| GD | *gd1c* | 1 | RM212-RM6703 | 2.12 | 0.35 | 11.08 |  |  |  |  |  |  |  |  |  |  |  |  |
| GD | *gd2* | 2 | RM5862-RM7355 |  |  |  |  |  |  | 2.20 | -0.66 | 18.56 |  |  |  |  |  |  |
| GD | *gd3a* | 3 | MRG5959-MRG2180 | |  |  |  |  |  |  |  |  | 2.32 | 0.34 | 15.07 |  |  |  |
| GD | *gd3b* | 3 | RM227-RM514 |  |  |  | 3.32 | 0.43 | 22.94 |  |  |  | 3.66 | 0.44 | 23.31 |  |  |  |
| GD | *gd4* | 4 | RM252-RM241 | 3.50 | 0.49 | 25.42 |  |  |  |  |  |  |  |  |  |  |  |  |
| GD | *gd6a* | 6 | RM589-RM584 |  |  |  |  |  |  | 2.28 | 0.62 | 20.98 |  |  |  |  |  |  |
| GD | *gd6b* | 6 | RM584-RM314 |  |  |  |  |  |  |  |  |  |  |  |  | 2.80 | 0.79 | 19.09 |
| GD | *gd7a* | 7 | RM11-RM3753 |  |  |  |  |  |  |  |  |  | 2.51 | 0.40 | 23.73 |  |  |  |
| GD | *gd7b* | 7 | RM3753-RM473A |  |  |  |  |  |  | 2.54 | 0.44 | 22.44 |  |  |  |  |  |  |
|  |  |  |  |  |  |  |  |  |  |  |  |  |  |  |  |  |  |  |
| YD | *yd1* | 1 | RM1247-RM6324 | 3.43 | -1.30 | 13.39 |  |  |  |  |  |  |  |  |  |  |  |  |
| YD | *yd2* | 2 | RM5916-RM535 |  |  |  |  |  |  |  |  |  |  |  |  | 2.62 | 1.31 | 11.14 |
| YD | *yd3* | 3 | RM3392-RM5925 |  |  |  | 2.24 | -0.87 | 8.66 | 2.41 | -0.87 | 7.61 | 4.41 | -1.42 | 15.50 |  |  |  |
| YD | *yd5* | 5 | RM3437-RM473B | 2.02 | 1.36 | 13.65 |  |  |  |  |  |  |  |  |  |  |  |  |
| YD | *yd7a* | 7 | RM3325-RM1253 |  |  |  | 2.44 | -1.15 | 9.22 |  |  |  |  |  |  |  |  |  |
| YD | *yd7b* | 7 | RM1253-RM3583 |  |  |  |  |  |  | 4.28 | -1.47 | 13.93 |  |  |  |  |  |  |
| YD | *yd8a* | 8 | RM152-MRG0270 |  |  |  | 2.54 | 1.47 | 13.52 |  |  |  |  |  |  |  |  |  |
| YD | *yd8b* | 8 | RM25-MRG2181 |  |  |  |  |  |  |  |  |  |  |  |  | 6.88 | -2.71 | 23.76 |

Note: a Chromosome number of the QTL detected in the study.

b Gca_1_, Gca_2_ and Gca_3_ represent the Gca data set derived from the BCRIL and two of the three TC populations TCP_1_/TCP_2_, TCP_1_/TCP_3_, TCP_2_/TCP_3_, respectively.
