# Supplementary material for: QTL Mapping of Combining Ability and Heterosis of Agronomic Traits in Rice Backcross Recombinant Inbred Lines and Hybrid Crosses
Source: PLoS One. 2012 Jan 26;7(1):e28463. doi: 10.1371/journal.pone.0028463 (PMC3266898; doi:10.1371/journal.pone.0028463)
Supplement: Table S6 — Main-effect QTL detected in BCRIL and Gca data sets. (DOC) [file pone.0028463.s006.doc]

Table S6 Main-effect QTL detected in BCRIL and Gca data sets

| Trait^a^ | QTL | Chr^b^ | Interval | BCRIL | | | Gca | | |
| --- | --- | --- | --- | --- | --- | --- | --- | --- | --- |
|  |  |  |  | LOD | A^c^ | *R*^2^ (%)^d^ | LOD | A^c^ | *R*^2^ (%)^d^ |
| PH | *ph3* | 3 | RM532-RM520 | - | - | - | 4.06 | -3.16 | 24.04 |
| PH | *ph4a* | 4 | RM185-RM273 | - | - | - | 2.19 | -1.65 | 6.90 |
| PH | *ph4b* | 4 | RM273-RM252 | 3.86 | 3.68 | 16.91 | - | - | - |
| PH | *ph7* | 7 | RM3583-RM7110 | - | - | - | 5.45 | -3.49 | 25.19 |
| PH | *ph8* | 8 | RM25-MRG2181 | 4.77 | -5.36 | 21.31 | 4.51 | -3.55 | 25.53 |
| PH | *ph12* | 12 | RM3717-RM19 | - | - | - | 3.07 | -1.84 | 11.25 |
|  |  |  |  |  |  |  |  |  |  |
| HD | *hd1* | 1 | RM151-RM8083 | 2.83 | -1.12 | 7.99 | - | - | - |
| HD | *hd3* | 3 | RM520-RM293 | 4.39 | -2.01 | 12.95 | - | - | - |
| HD | *hd6* | 6 | RM584-RM314 | 5.43 | 3.17 | 17.37 | - | - | - |
| HD | *hd7* | 7 | RM1253-RM3583 | 9.89 | -2.98 | 36.92 | 4.07 | -2.83 | 24.18 |
| HD | *hd8* | 8 | RM25-MRG2181 | 5.45 | -2.70 | 22.80 | 4.87 | -3.24 | 21.65 |
|  |  |  |  |  |  |  |  |  |  |
| TP | *tp1a* | 1 | RM462-RM1247 | 3.03 | 0.51 | 18.78 | - | - | - |
| TP | *tp1b* | 1 | RM151-RM8083 | - | - | - | 3.07 | 1.36 | 33.40 |
| TP | *tp2a* | 2 | RM5862-RM7355 | - | - | - | 2.93 | 1.69 | 28.33 |
| TP | *tp2b* | 2 | RM6318-RM526 | 2.47 | -0.49 | 12.94 | - | - | - |
| TP | *tp4a* | 4 | RM307-RM261 | 3.42 | 0.72 | 21.28 | - | - | - |
| TP | *tp4b* | 4 | RM261-R4M30 | - | - | - | 2.74 | 1.39 | 25.89 |
| TP | *tp5* | 5 | RM3321-RM480 | 5.52 | 0.86 | 35.69 | - | - | - |
| TP | *tp6* | 6 | RM589-RM584 | - | - | - | 3.17 | -1.66 | 23.97 |
| TP | *tp11a* | 11 | RM6327-RM1812 | 2.33 | -0.64 | 21.59 | - | - | - |
| TP | *tp12a* | 12 | MRG0986-RM3331 | - | - | - | 2.21 | 0.87 | 14.93 |
|  |  |  |  |  |  |  |  |  |  |
| PL | *pl1a* | 1 | RM462-RM1247 | 2.54 | 10.97 | 15.00 | - | - | - |
| PL | *pl2a* | 2 | RM5862-RM7355 | - | - | - | 3.03 | 47.80 | 23.45 |
| PL | *pl2b* | 2 | RM6318-RM526 | 3.77 | -15.60 | 22.92 | - | - | - |
| PL | *pl3a* | 3 | RM569-RM3392 | - | - | - | 2.41 | 47.88 | 23.47 |
| PL | *pl3b* | 3 | MRG5959-MRG2180 | 2.05 | 12.72 | 15.18 | - | - | - |
| PL | *pl5a* | 5 | RM3295-RM7081 | 3.54 | 16.91 | 27.58 | - | - | - |
| PL | *pl6b* | 6 | RM121-RM6071 | - | - | - | 5.55 | 51.29 | 56.17 |
| PL | *pl7* | 7 | RM3583-RM7110 | - | - | - | 4.02 | 42.26 | 21.45 |
| PL | *pl8* | 8 | RM152-MRG0270 | - | - | - | 3.14 | 51.20 | 31.05 |
|  |  |  |  |  |  |  |  |  |  |
| FGPP | *fgpp1a* | 1 | RM488-RM246 | 4.45 | -151.01 | 41.14 | - | - | - |
| FGPP | *fgpp1b* | 1 | RM6666-RM212 | - | - | - | 2.19 | -140.04 | 11.68 |
| FGPP | *fgpp3* | 3 | MRG5959-MRG2180 | - | - | - | 3.62 | 203.69 | 23.80 |
| FGPP | *fgpp4* | 4 | RM252-RM241 | - | - | - | 2.16 | -94.40 | 9.22 |
| FGPP | *fgpp5* | 5 | RM7081-RM3321 | 2.16 | 101.59 | 14.39 | - | - | - |
| FGPP | *fgpp6* | 6 | RM584-RM314 | - | - | - | 2.80 | 216.72 | 24.88 |
| FGPP | *fgpp7* | 7 | RM51-RM3325 | - | - | - | 2.97 | 202.00 | 26.30 |
|  |  |  |  |  |  |  |  |  |  |
| SS | *ss2a* | 2 | RM5862-RM7355 | 2.37 | 4.41 | 12.06 | - | - | - |
| SS | *ss2b* | 2 | RM5699-RM324 | - | - | - | 3.96 | -3.79 | 25.09 |
| SS | *ss3* | 3 | RM3392-RM5925 | 5.90 | -5.73 | 36.11 | - | - | - |
| SS | *ss5* | 5 | RM440-RM3575 | - | - | - | 3.55 | 3.25 | 27.53 |
| SS | *ss7* | 7 | RM473A-MRG2555 | - | - | - | 2.19 | -2.22 | 12.44 |
| SS | *ss9* | 9 | RM553-RM215 | 2.10 | -3.63 | 15.58 | - | - | - |
| SS | *ss11* | 11 | RM1812-MRG5615 | 4.49 | -4.44 | 30.44 | - | - | - |
|  |  |  |  |  |  |  |  |  |  |
| GPP | *gpp6* | 6 | RM121-RM6071 | - | - | - | 4.08 | 954.03 | 58.73 |
| GPP | *gpp7* | 7 | RM3583-RM7110 | - | - | - | 2.16 | 323.63 | 14.83 |
|  |  |  |  |  |  |  |  |  |  |
| SPP | *spp1* | 1 | RM462-RM1247 | 3.23 | -8.49 | 13.62 | - | - | - |
| SPP | *spp2* | 2 | RM7355-RM5699 | - | - | - | 2.31 | -11.01 | 14.76 |
| SPP | *spp4* | 4 | RM252-RM241 | 4.53 | 9.32 | 19.40 | - | - | - |
| SPP | *spp6* | 6 | RM314-RM50 | - | - | - | 3.43 | 12.98 | 17.84 |
| SPP | *spp11* | 11 | RM202-RM287 | 2.56 | -8.77 | 11.01 | - | - | - |
|  |  |  |  |  |  |  |  |  |  |
| GD | *gd1a* | 1 | RM462-RM1247 | 2.34 | -0.36 | 14.19 | - | - | - |
| GD | *gd1b* | 1 | RM283-RM151 | - | - | - | 3.66 | -0.46 | 25.00 |
| GD | *gd3* | 3 | RM227-RM514 | - | - | - | 3.32 | 0.43 | 22.94 |
| GD | *gd4* | 4 | RM252-RM241 | 3.50 | 0.49 | 25.42 | - | - | - |
|  |  |  |  |  |  |  |  |  |  |
| YD | *yd1* | 1 | RM1247-RM6324 | 3.43 | -1.30 | 13.39 | - | - | - |
| YD | *yd3* | 3 | RM3392-RM5925 | - | - | - | 2.24 | -0.87 | 8.66 |
| YD | *yd5* | 5 | RM3437-RM473B | 2.02 | 1.36 | 13.65 | - | - | - |
| YD | *yd7* | 7 | RM3325-RM1253 | - | - | - | 2.44 | -1.15 | 9.22 |
| YD | *yd8* | 8 | RM152-MRG0270 | - | - | - | 2.54 | 1.47 | 13.52 |

a For a description of agronomic traits see materials and methods.

b Chromosome number of the QTL detected in the study.

c A represents additive effect of main-effect QTL. It should be noted that, when the genotype frequency *p=q=*1/2, a QTL identified in the Gca data set showed only additive effects. If not, additive and dominance effects were confounded.

d *R*^2^ represents the phenotypic variation explained by main-effect QTL.
