# Supplementary material for: QTL Mapping of Combining Ability and Heterosis of Agronomic Traits in Rice Backcross Recombinant Inbred Lines and Hybrid Crosses
Source: PLoS One. 2012 Jan 26;7(1):e28463. doi: 10.1371/journal.pone.0028463 (PMC3266898; doi:10.1371/journal.pone.0028463)
Supplement: Table S7 — Main-effect QTL detected in Sca and Hmp data sets of three TC populations. (DOC) [file pone.0028463.s007.doc]

Table S7 Main-effect QTL detected in Sca and Hmp data sets of three TC populations

| Trait^a^ | QTL | Chr^b^ | Interval | Sca893s | | | Hmp893s | | | Sca888s | | | Hmp888s | | | Sca64s | | | Hmp64s | | |
| --- | --- | --- | --- | --- | --- | --- | --- | --- | --- | --- | --- | --- | --- | --- | --- | --- | --- | --- | --- | --- | --- |
|  |  |  |  | LOD | D^c^ | *R*^2^(%)^d^ | LOD | D^c^ | *R*^2^(%)^d^ | LOD | D^c^ | *R*^2^(%)^d^ | LOD | D^c^ | *R*^2^(%)^d^ | LOD | D^c^ | *R*^2^(%)^d^ | LOD | D^c^ | *R*^2^(%)^d^ |
| PH | *D-ph4a* | 4 | RM261-R4M30 | - | - | - | - | - | - | - | - | - | - | - | - | 4.46 | -1.93 | 22.24 | - | - | - |
| PH | *D-ph4b* | 4 | RM185-RM273 | - | - | - | - | - | - | - | - | - | - | - | - | - | - | - | 3.92 | -3.08 | 18.81 |
| PH | *D-ph7* | 7 | RM3583-RM7110 | - | - | - | - | - | - | - | - | - | - | - | - | 6.64 | 2.94 | 31.79 | - | - | - |
| PH | *D-ph8* | 8 | RM25-MRG2181 | 7.05 | 2.81 | 35.37 | - | - | - | - | - | - | - | - | - | - | - | - | - | - | - |
|  |  |  |  |  |  |  |  |  |  |  |  |  |  |  |  |  |  |  |  |  |  |
| HD | *D-hd2* | 2 | RM7355-RM5699 | - | - | - | - | - | - | 5.87 | 2.48 | 27.79 | - | - | - | - | - | - | - | - | - |
| HD | *D-hd3* | 3 | R3M37-RM532 | - | - | - | - | - | - | 3.05 | 1.90 | 13.67 | - | - | - | - | - | - | - | - | - |
| HD | *D-hd4a* | 4 | R4M30-RM185 | - | - | - | - | - | - | 3.68 | 1.91 | 18.33 | - | - | - | - | - | - | - | - | - |
| HD | *D-hd4b* | 4 | RM252-RM241 | - | - | - | - | - | - | - | - | - | - | - | - | 5.00 | 2.57 | 28.94 | - | - | - |
| HD | *D-hd6* | 6 | RM584-RM314 | - | - | - | - | - | - | 4.03 | -3.37 | 15.69 | 3.41 | -5.35 | 13.91 | - | - | - | - | - | - |
| HD | *D-hd8* | 8 | RM25-MRG2181 | 4.9 | 2.23 | 23.19 | - | - | - | - | - | - | 3.16 | -3.93 | 12.78 | - | - | - | 3.11 | -2.54 | 17.69 |
| HD | *D-hd9* | 9 | RM6839B-RM257 | 3.68 | 1.34 | 20.73 | - | - | - | - | - | - | - | - | - | - | - | - | - | - | - |
|  |  |  |  |  |  |  |  |  |  |  |  |  |  |  |  |  |  |  |  |  |  |
| TP | *D-tp2* | 2 | RM530-RM5916 | - | - | - | - | - | - | - | - | - | 4.29 | -1.47 | 28.17 | - | - | - | - | - | - |
| TP | *D-tp3a* | 3 | RM569-RM3392 | 4.12 | -1.01 | 26.54 | - | - | - | - | - | - | - | - | - | - | - | - | - | - | - |
| TP | *D-tp3b* | 3 | MRG2180-RM6733 | - | - | - | - | - | - | - | - | - | - | - | - | 3.16 | 0.43 | 16.85 | - | - | - |
| TP | *D-tp3c* | 3 | RM293-RM227 | 4.7 | -0.75 | 39.03 | - | - | - | 4.21 | 0.51 | 20.14 | - | - | - | - | - | - | - | - | - |
| TP | *D-tp4a* | 4 | R4M30-RM185 | - | - | - | - | - | - | - | - | - | - | - | - | - | - | - | 4.61 | -1.54 | 37.51 |
| TP | *D-tp4b* | 4 | RM303-MRG0113 | - | - | - | - | - | - | 4.99 | -0.76 | 24.90 | - | - | - | - | - | - | - | - | - |
| TP | *D-tp5* | 5 | RM7081-RM3321 | - | - | - | 3.63 | -1.60 | 24.60 | - | - | - | - | - | - | - | - | - | - | - | - |
| TP | *D-tp6a* | 6 | RM314-RM50 | - | - | - | - | - | - | - | - | - | 4.43 | 1.93 | 33.93 | - | - | - | - | - | - |
| TP | *D-tp6b* | 6 | RM6071-RM30 | 3.31 | 0.80 | 22.18 | - | - | - | - | - | - | - | - | - | - | - | - | - | - | - |
| TP | *D-tp6c* | 6 | RM30-RM494 | - | - | - | - | - | - | - | - | - | - | - | - | 5.23 | -0.62 | 30.92 | - | - | - |
|  |  |  |  |  |  |  |  |  |  |  |  |  |  |  |  |  |  |  |  |  |  |
| PL | *D-pl1* | 1 | RM1287-RM306 | - | - | - | - | - | - | 4.08 | 14.75 | 29.29 |  |  |  |  |  |  |  |  |  |
| PL | *D-pl3* | 3 | MRG5959-MRG2180 | - | - | - | - | - | - |  |  |  |  |  |  | 4.81 | 18.76 | 29.52 |  |  |  |
| PL | *D-pl4* | 4 | RM185-RM273 | 2.13 | -12.54 | 20.59 | - | - | - | 4.85 | 15.97 | 40.17 | - | - | - | - | - | - | - | - | - |
| PL | *D-pl6a* | 6 | RM589-RM584 | - | - | - | - | - | - | - | - | - | 3.81 | -75.01 | 24.20 | - | - | - | - | - | - |
| PL | *D-pl6b* | 6 | RM584-RM314 | - | - | - | - | - | - | - | - | - | - | - | - | - | - | - | 4.31 | 79.90 | 49.86 |
| PL | *D-pl6c* | 6 | RM30-RM494 | - | - | - | - | - | - | - | - | - | - | - | - | 4.53 | -14.75 | 27.42 | - | - | - |
| PL | *D-pl11* | 11 | RM206-RM254 | - | - | - | - | - | - | 3.58 | 10.89 | 22.68 | 3.63 | 32.24 | 30.47 | - | - | - | - | - | - |
|  |  |  |  |  |  |  |  |  |  |  |  |  |  |  |  |  |  |  |  |  |  |
| FGPP | *D-fgpp1a* | 1 | RM151-RM8083 | 4.12 | -128.51 | 30.61 | - | - | - | - | - | - | - | - | - | - | - | - | - | - | - |
| FGPP | *D-fgpp1b* | 1 | RM6703-RM3362 | - | - | - | - | - | - | 5.37 | -115.88 | 27.72 | - | - | - | - | - | - | - | - | - |
| FGPP | *D-fgpp2* | 2 | RM530-RM5916 | - | - | - | - | - | - | 8.63 | -140.36 | 59.95 | - | - | - | - | - | - | - | - | - |
| FGPP | *D-fgpp3a* | 3 | MRG5959-MRG2180 | - | - | - | - | - | - |  |  |  | - | - | - | - | - | - | 3.60 | 384.65 | 45.42 |
| FGPP | *D-fgpp3b* | 3 | RM532-RM520 | - | - | - | - | - | - | 4.78 | -105.98 | 21.5 | - | - | - | - | - | - | - | - | - |
| FGPP | *D-fgpp4* | 4 | RM261-R4M30 | - | - | - | 3.42 | -141.69 | 15.41 | - | - | - | - | - | - | - | - | - | - | - | - |
| FGPP | *D-fgpp5* | 5 | RM3437-RM473B | - | - | - | - | - | - | 3.60 | -82.61 | 20.13 | - | - | - | - | - | - | - | - | - |
| FGPP | *D-fgpp6* | 6 | RM584-RM314 | - | - | - | - | - | - | - | - | - | 5.35 | 370.28 | 55.03 | - | - | - | - | - | - |
| FGPP | *D-fgpp7a* | 7 | RM3325-RM1253 | - | - | - | 5.67 | 212.58 | 31.97 | - | - | - | - | - | - | - | - | - | - | - | - |
| FGPP | *D-fgpp7b* | 7 | RM11-RM3753 | - | - | - | - | - | - | 3.75 | -106.12 | 21.51 | - | - | - | - | - | - | - | - | - |
| FGPP | *D-fgpp8* | 8 | MRG2181-RM483 | - | - | - | 3.46 | -194.67 | 15.62 | - | - | - | - | - | - | - | - | - | - | - | - |
| FGPP | *D-fgpp12* | 12 | RM5338-YSB1 | - | - | - | - | - | - | 3.21 | -106.20 | 21.51 | - | - | - | - | - | - | - | - | - |
|  |  |  |  |  |  |  |  |  |  |  |  |  |  |  |  |  |  |  |  |  |  |
| SS | *D-ss1* | 1 | RM6703-RM3362 | - | - | - | - | - | - | - | - | - | - | - | - | - | - | - | 3.56 | 6.53 | 17.41 |
| SS | *D-ss2a* | 2 | RM5862-RM7355 | - | - | - | 4.97 | -6.84 | 36.67 | - | - | - | - | - | - | - | - | - | - | - | - |
| SS | *D-ss2b* | 2 | RM5916-RM535 | - | - | - | - | - | - | 3.08 | -2.32 | 14.02 | - | - | - | - | - | - | - | - | - |
| SS | *D-ss3a* | 3 | RM5925-RM218 | - | - | - | - | - | - | - | - | - | 4.18 | 5.21 | 29.2 | - | - | - | - | - | - |
| SS | *D-ss3b* | 3 | R3M37-RM532 | - | - | - | - | - | - | - | - | - | - | - | - | - | - | - | 3.89 | 4.81 | 19.02 |
| SS | *D-ss3c* | 3 | RM293-RM227 | - | - | - | - | - | - | 4.01 | -3.34 | 22.86 | - | - | - | - | - | - | - | - | - |
| SS | *D-ss4a* | 4 | RM261-R4M30 | - | - | - | - | - | - | - | - | - | - | - | - | 3.37 | 2.49 | 15.51 | - | - | - |
| SS | *D-ss4b* | 4 | RM185-RM273 | - | - | - | - | - | - | - | - | - | 3.61 | -4.92 | 27.71 | - | - | - | - | - | - |
| SS | *D-ss5a* | 5 | RM473B-RM440 | - | - | - | - | - | - | - | - | - | - | - | - | - | - | - | 5.94 | 5.59 | 33.1 |
| SS | *D-ss5b* | 5 | RM3321-RM480 | - | - | - | - | - | - | 3.08 | 2.27 | 14.95 | - | - | - | - | - | - | - | - | - |
| SS | *D-ss6a* | 6 | RM589-RM584 | - | - | - | - | - | - | - | - | - | - | - | - | 4.57 | -4.36 | 23.95 | - | - | - |
| SS | *D-ss6b* | 6 | RM121-RM6071 | - | - | - | - | - | - | 3.18 | -3.34 | 22.81 | - | - | - | - | - | - | - | - | - |
| SS | *D-ss7* | 7 | RM3325-RM1253 | - | - | - | - | - | - | 4.06 | -3.39 | 23.71 | - | - | - | - | - | - | - | - | - |
| SS | *D-ss8* | 8 | RM25-MRG2181 | - | - | - | - | - | - | 5.91 | 5.2 | 34.43 | - | - | - | 3.2 | -2.75 | 14.51 | - | - | - |
| SS | *D-ss12a* | 12 | RM3472-RM7003 | - | - | - | - | - | - | - | - | - | - | - | - | - | - | - | 4.69 | -4.26 | 24.08 |
| SS | *D-ss12b* | 12 | RM5338-YSB1 | - | - | - | - | - | - | 4.53 | -3.18 | 25.02 | - | - | - | - | - | - | - | - | - |
|  |  |  |  |  |  |  |  |  |  |  |  |  |  |  |  |  |  |  |  |  |  |
| GPP | *D-gpp1* | 1 | RM572-RM1287 | 3.81 | -175.59 | 30.45 | - | - | - |  |  |  |  |  |  |  |  |  |  |  |  |
| GPP | *D-gpp2* | 2 | RM530-RM5916 | - | - | - | - | - | - | 5.36 | -130.52 | 29.49 | - | - | - | - | - | - | - | - | - |
| GPP | *D-gpp3* | 3 | MRG5959-MRG2180 | - | - | - | - | - | - | - | - | - | 3.57 | -258.44 | 16.87 | 4.46 | 176.46 | 28.97 | - | - | - |
| GPP | *D-gpp6a* | 6 | RM584-RM314 | - | - | - | - | - | - | - | - | - | 8.78 | 556.52 | 58.95 | - | - | - | 3.64 | 467.66 | 36.46 |
| GPP | *D-gpp6b* | 6 | RM121-RM6071 | - | - | - | - | - | - | - | - | - | - | - | - | - | - | - | 4.48 | 789.55 | 48.59 |
| GPP | *D-gpp7* | 7 | RM3325-RM1253 | - | - | - | 3.86 | 360.32 | 25.28 | - | - | - | - | - | - | - | - | - | - | - | - |
| GPP | *D-gpp10* | 10 | RM6364-MRG4348 | - | - | - | - | - | - | - | - | - | - | - | - | - | - | - | 3.63 | -261.85 | 23.82 |
|  |  |  |  |  |  |  |  |  |  |  |  |  |  |  |  |  |  |  |  |  |  |
| SPP | *D-spp1a* | 1 | RM151-RM8083 | - | - | - | 4.97 | -19.26 | 33.38 | - | - | - | - | - | - | - | - | - | - | - | - |
| SPP | *D-spp1b* | 1 | RM1287-RM306 | - | - | - | - | - | - | 3.79 | 7.96 | 19.00 | - | - | - | - | - | - | - | - | - |
| SPP | *D-spp3a* | 3 | RM569-RM3392 | - | - | - | - | - | - | 4.27 | -12.90 | 16.03 | - | - | - | - | - | - | - | - | - |
| SPP | *D-spp3b* | 3 | MRG5959-MRG2180 | - | - | - | - | - | - | - | - | - | - | - | - | - | - | - | 3.70 | 14.78 | 39.08 |
| SPP | *D-spp3c* | 3 | RM227-RM514 | - | - | - | - | - | - |  |  |  | - | - | - | - | - | - | 4.16 | 14.89 | 33.53 |
| SPP | *D-spp4* | 4 | RM5688-RM307 | - | - | - | - | - | - | 5.64 | 7.98 | 19.05 | - | - | - | - | - | - | - | - | - |
| SPP | *D-spp6a* | 6 | RM589-RM584 | - | - | - | - | - | - | 4.49 | 7.45 | 16.81 | - | - | - | - | - | - | - | - | - |
| SPP | *D-spp6b* | 6 | RM314-RM50 | - | - | - | - | - | - | - | - | - | - | - | - | 4.01 | 25.39 | 22.71 | - | - | - |
| SPP | *D-spp7a* | 7 | RM3325-RM1253 | - | - | - | - | - | - | 5.44 | 7.45 | 19.26 | - | - | - | - | - | - | - | - | - |
| SPP | *D-spp7b* | 7 | RM11-RM3753 | 3.71 | 10.40 | 29.90 | - | - | - | - | - | - | - | - | - | 5.42 | -27.88 | 33.91 | - | - | - |
| SPP | *D-spp8* | 8 | RM25-MRG2181 | - | - | - | - | - | - | 3.29 | -9.58 | 13.43 | - | - | - | - | - | - | - | - | - |
| SPP | *D-spp11a* | 11 | RM1812-MRG5615 | - | - | - | - | - | - | 3.86 | 7.46 | 19.23 | - | - | - | - | - | - | - | - | - |
| SPP | *D-spp11b* | 11 | RM206-RM254 | - | - | - | - | - | - | - | - | - | - | - | - | 3.07 | -19.72 | 16.29 | - | - | - |
| SPP | *D-spp12* | 12 | RM5338-YSB1 | - | - | - | - | - | - | 4.97 | -8.40 | 27.66 | - | - | - | - | - | - | - | - | - |
|  |  |  |  |  |  |  |  |  |  |  |  |  |  |  |  |  |  |  |  |  |  |
| GD | *D-gd1* | 1 | RM572-RM1287 | 4.04 | -0.33 | 24.69 | - | - | - | - | - | - | - | - | - | - | - | - | - | - | - |
| GD | *D-gd2* | 2 | RM5916-RM535 | - | - | - | - | - | - | 6.57 | -0.29 | 36.54 | - | - | - | - | - | - | - | - | - |
| GD | *D-gd3a* | 3 | RM7197-MRG5959 | - | - | - | 3.33 | 0.67 | 20.74 | - | - | - | - | - | - | - | - | - | - | - | - |
| GD | *D-gd3b* | 3 | RM6733-MRG4626 | - | - | - | - | - | - | - | - | - | - | - | - | 3.70 | -0.31 | 22.44 | - | - | - |
| GD | *D-gd3c* | 3 | RM227-RM514 | - | - | - | - | - | - | - | - | - | 3.85 | 0.56 | 23.37 | - | - | - | - | - | - |
| GD | *D-gd4a* | 4 | RM5688-RM307 | - | - | - | - | - | - | - | - | - | - | - | - | 3.16 | -0.29 | 29.59 | - | - | - |
| GD | *D-gd4b* | 4 | RM303-MRG0113 | - | - | - | - | - | - | - | - | - | - | - | - | - | - | - | 4.46 | -0.53 | 31.29 |
| GD | *D-gd9* | 9 | RM215-RM245 | - | - | - | - | - | - | - | - | - | - | - | - | - | - | - | - | - | - |
| GD | *D-gd10a* | 10 | RM6364-MRG4348 | - | - | - | - | - | - | 5.06 | -0.29 | 27.82 | - | - | - | - | - | - | - | - | - |
| GD | *D-gd10b* | 10 | RM5689-RM1873 | - | - | - | 6.58 | -0.81 | 38.32 | - | - | - | - | - | - | - | - | - | - | - | - |
| GD | *D-gd11* | 11 | RM206-RM254 | - | - | - | - | - | - | - | - | - | - | - | - | 4.57 | -0.38 | 28.32 | - | - | - |
| GD | *D-gd12* | 12 | RM5338-YSB1 | - | - | - | - | - | - | - | - | - | 3.39 | -0.50 | 23.37 | - | - | - | - | - | - |
|  |  |  |  |  |  |  |  |  |  |  |  |  |  |  |  |  |  |  |  |  |  |
| YD | *D-yd3* | 3 | RM569-RM3392 | - | - | - | - | - | - | 4.10 | 1.96 | 27.84 | - | - | - | - | - | - | - | - | - |
| YD | *D-yd7* | 7 | RM1253-RM3583 | - | - | - | 3.38 | -2.04 | 11.13 | - | - | - | - | - | - | - | - | - | - | - | - |
| YD | *D-yd11* | 11 | RM254-RM224 | 4.3 | -1.71 | 19.03 | - | - | - | 3.18 | 1.46 | 11.69 | - | - | - | - | - | - | - | - | - |

a For a description of agronomic traits see materials and methods.

^b^ Chromosome number of the QTL detected in the study.

^c^ D represents dominance effect of main-effect QTL. It should be noted that the QTL detected in Hmp and Sca data set show dominant effect, only if there are two alleles at each locus.

^d^ *R*^2^ represents the phenotypic variation explained by main-effect QTL.
